# A Model-Based Approach for Dynamic Characterisation of Force Transducers with Impact Hammers

**DOI:** 10.3390/s26185972

**Published:** 2026-09-21

**Authors:** Gianmarco Battista, Stefano Pavoni, Francescantonio Lucà, Marta Berardengo, Marcello Vanali

**Affiliations:** 1 Department of Engineering for Industrial Systems and Technologies, University of Parma, 43124 Parma, Italy; stefano.pavoni@unipr.it (S.P.); marcello.vanali@unipr.it (M.V.); 2 Department of Mechanical Engineering, Politecnico di Milano, Via La Masa, 34, 20156 Milan, Italy; 3 Department of Mechanical, Energy, Management and Transportation Engineering, University of Genova, 16145 Genova, Italy; marta.berardengo@unige.it

**Keywords:** force transducers, load cells, dynamic force, impulse force, dynamic calibration, model-based characterisation, frequency domain characterisation, sensor characterisation

## Abstract

This paper presents a method for the dynamic characterisation of load cells and force transducers designed to be straightforward to implement and based on instrumentation commonly used in structural dynamics testing. Impact hammers simultaneously provide an impulsive force to the sensor under test and measure the actual input to estimate the frequency response function. The bandwidth of the sensor is evaluated using an analytical single- or multi-degree-of-freedom frequency-domain model that is fitted to the experimental frequency response function using a Non-Linear Least-Squares approach. This paper provides guidelines for selecting the model complexity and the fitting frequency range and demonstrates the procedure on an experimental strain-gauge load cell. For the investigated transducer, the identified first natural frequency was 481.4 Hz, resulting in bandwidths of 47.9 Hz, 105.1 Hz, and 154.2 Hz for allowable deviations from the static sensitivity of 1%, 5%, and 10%, respectively. Moreover, for the single-degree-of-freedom case, the effect of masses added under operating conditions is modelled, with a worst-case relative error below 1% in the prediction of the natural frequency for the validation measurements, thus enabling the actual bandwidth to be estimated.

## 1. Introduction

Force is a physical quantity measured in a wide range of industrial applications and research fields. The sensors used for this purpose are commonly referred to as load cells and/or force transducers. One of the most commonly used types is the strain-gauge load cell, due to its low cost, ease of use, and the flexibility in designing novel and advantageous force/torque sensor configurations [1,2]. Over the years, this type of transducer has increasingly been used to measure dynamic forces, thus raising several challenges in its characterisation and use. The current standards provide procedures for static calibration [3], or for particular cases of dynamic calibration [4]. However, the latter standard solely covers sinusoidal forces with constant amplitude and is applicable to dynamic testing systems operating away from resonances. Instead, only some guidelines are provided for dynamic force measuring devices [5]. Both Kobusch [6] and Fujii [7] highlighted the lack of a general standard for dynamic force calibration due to the infinite “shapes” of time-varying forces. Different calibration methods have been proposed that rely on three types of dynamic excitation: impact force, step force, and sinusoidal force [8,9].

In [10,11], dynamic calibration is performed by applying a known impact force to the transducer under calibration through the collision of a known mass with the sensor. The underlying principle is that the change in momentum of the mass is related to the input force. The instantaneous velocity of the mass is measured by means of Laser-Doppler Interferometry (LDI). A pneumatic linear bearing is used to ensure linear motion of the mass with sufficiently low friction. In [12], a tailored device for primary shock force calibration up to 250 kN is presented.

A similar dynamic calibration method for force transducers is represented by the step response. In [13], a force step is generated using a mass suspended above the transducer by a stainless-steel wire. By cutting the wire, the mass is released. In this case too, a pneumatic linear bearing is used to ensure vertical motion and reduce friction. Again, an optical interferometer measures the mass velocity from which the acceleration is calculated and, finally, the force. However, in this case, the transducer response is affected by the mass, which remains coupled to the sensor.

A further class of methods implements dynamic calibration of force transducers using sinusoidal excitations. Some specific guidelines are reported in [14]. The method proposed in [15] uses a steel spring which connects the transducer to a known mass: the oscillating force is triggered by manually hitting the mass using a hammer. The input force is calculated from the velocity of the mass measured by means of LDI. In [16], the force transducer is mounted on an electro-dynamic shaker system through a metal spring and equipped with a known mass. The shaker is activated using a sinusoidal signal with a known frequency. The excitation force is calculated from the acceleration of the mass, which is again derived from the velocity measured by LDI. Specific aspects of the dynamic calibration of force transducers using the sinusoidal method are investigated in detail in [17]. This study focuses on the importance of acceleration measurement via laser vibrometer and how factors like the mass block’s size, density, and Young’s modulus influence acceleration distribution, which in turn affects the calibration accuracy. The study conducted in [18] provides a detailed comparison between static, continuous and dynamic calibration of a 200 N high-precision strain-gauge force transducer. Typically, the uncertainties under continuous and dynamic conditions are one or two orders of magnitude higher than under static conditions (<0.1%).

Most of the methods mentioned above require expensive instruments and devices to provide the input force or to measure the physical quantities. Another cause for concern regarding the dynamic characterisation of force transducers is whether a calibration carried out using a specific type of time-varying force remains valid when, under operating conditions, the transducer is subjected to a different force signal. For this reason, Fujii [7] highlights the importance of having access to the information provided by manufacturers on the dynamic characteristics of the sensors. Knowledge of the dynamic characteristics of a sensor enables strategies such as dynamic compensation of its response [19].

A step forward in this direction is made in [20], where a model-based calibration is proposed using impact excitation. A lumped linear model with different mass-spring-damper (MSD) systems is adopted to represent the characteristics of force transducers. However, the model parameters are estimated by fitting the measured time-domain signals, which makes the estimation procedure sensitive to the fitting interval, the actual shape of the force pulse, initial conditions and the noise affecting the decaying response.

Another important aspect to consider is the classification of dynamic calibration into primary and secondary methods, based on how metrological traceability of the input force is guaranteed [5]. For primary methods, the input force is generated by means of accelerated masses according to Newton’s second law [21]. Most of the studies cited above adopt primary calibration methods, independently of the shape of the input force signal. Instead, for secondary methods, the same input force is supplied concurrently to the sensor under calibration and to a reference sensor [22,23], such as impact hammers (often referred to as “modal hammers”) or automated impulse force generators [24]. These reference transducers can in turn be calibrated using a primary method [25].

The overview presented so far highlights a scenario where no practical, well-established standard for dynamic calibration exists. Therefore, further developments in this direction are desirable for two reasons. Firstly, access to a traceable reference can be facilitated for laboratories and industrial users. In fact, a reference force transducer can be easily portable and potentially cheaper than the instrumentation required to perform a primary calibration. Secondly, a portable reference instrument enables the dynamic calibration of force transducers in situ [26]. This would offer several advantages, such as the possibility of calibrating a sensor in its actual installation and measurement chain, i.e., taking into account the actual mechanical constraints, accessories, electronics, signal processing, etc.

This paper introduces a technique for the dynamic characterisation of force transducers that is suitable for a wide range of sensor configurations and requires only a basic measurement setup. The aim is to provide a method for the quick and practical characterisation of force transducers, but which can also be adopted for a proper dynamic calibration when using an already calibrated reference sensor. Moreover, this method can be applied both in laboratory and in situ characterisation of force sensors. A bending-beam strain-gauge load cell is used throughout the paper as an example in the characterisation procedure.

The basic hypothesis is that any system can be advantageously characterised in the frequency domain by assuming that it behaves as a Linear Time-Invariant system. For this reason, the proposed calibration method relies on the concept of frequency response function (FRF). The load cell FRF can be estimated from impact tests [27]. The impulsive force is generated by an impact hammer [28], while the embedded piezo-force transducer directly measures the dynamic input force supplied to the load cell being calibrated. The core idea of the approach is to represent the experimental FRF with a single- or multi-degree-of-freedom (DOF) frequency-domain analytical model, where each DOF is a different MSD system. The parameters can be estimated by means of Non-Linear Least-Squares (NLLS) [29]. The model-based dynamic calibration approach has already been demonstrated to be promising in [20,30]. However, the approach proposed in the present paper further exploits the advantages of working in the frequency domain. The first is the possibility to evaluate the quality of the FRF estimate by means of the coherence function [31], which allows the identification of excessive noise in the measurements and non-linearities in the system under test. The second advantage is the possibility of directly observing resonances in the modal response of the sensor, instead of observing ringing in the time-domain transducer response.

Thereafter, the model parameters (natural frequencies, damping ratios, etc.) are used to construct a synthetic FRF of the load cell, enabling a more precise evaluation of the sensor bandwidth, which is one of the most useful parameters in practical applications. Other key aspects of this approach need to be highlighted. The first is that the estimate of dynamic parameters can be performed independently of the static calibration of the load cell. The second is that the method addresses the main limitation of piezoelectric force transducers embedded in impact hammers, which are unable to measure input force components close to 0 Hz and lead to a biased FRF estimate at those frequencies. The calibration technique presented in this paper effectively circumvents this issue.

The paper is organised as follows. Section 2 briefly introduces the theoretical frequency-domain model of the load cell adopted for the characterisation. Section 3 describes the measurement setup and the FRF estimation method. Section 4 describes the procedure used to fit the analytical model to the measured FRF and employs the Monte Carlo Method (MCM) to investigate representative scenarios and derive practical guidelines for the fitting step. Section 5 presents the dynamic characterisation procedure based on impact-hammer testing and demonstrates its application to an experimental case. Section 6 compares the results with those obtained using the stepped-sine method. Section 7 presents a simple model for predicting the effect of added mass on the load cell bandwidth under operating conditions. Finally, Section 8 summarises the main conclusions.

## 2. Analytical Frequency-Domain Model of Load Cells for Dynamic Characterisation and Bandwidth Evaluation

The dynamical behaviour of a force transducer can be represented in terms of the relationship between the input force applied at the loading point and the output signal returned by the sensor. The latter can be an electrical signal or directly a force signal, if the static calibration has already been applied. A common hypothesis in dynamic characterisation of mechanical structures is that the system is LTI [30]. The linearity of the system implies that the input is amplified (or attenuated) and phase-shifted at each frequency independently of the others [31]. Under such conditions, the transducer characteristics can be represented by its FRF. In this work, the transducer response is considered in terms of force readings; therefore, the input-output relationship is expressed by the non-dimensional FRF ([N]/[N]), often referred to as “transmissibility”. This represents the dynamic sensitivity, i.e., the deviation from static sensitivity.

Strain-gauge load cells or other force transducers are mechanical dynamic systems that can be modelled as multi-DOF lumped-parameter systems comprising masses, springs, and dampers [6,31]. Each DOF is an MSD system. The analytical model of the load cell FRF used for dynamic characterisation purposes is represented by:

(1) $$H\left(f\right)=\sum_{i=1}^{D}\left[\frac{S_{i}}{1-{\left(\frac{f}{f_{ni}}\right)}^{2}+j2\zeta_{i}\left(\frac{f}{f_{ni}}\right)}\right]+R_{\mathrm{hi}}$$

where *D* is the number of modes, or equivalently the number of DOFs, considered in the model; *f* is the temporal frequency and j is the imaginary unit. Each mode, denoted by the subscript *i*, is determined by three parameters:

- $f_{ni}$ is the natural frequency;
- $S_{i}$ is the contribution of the *i*-th mode to the static sensitivity;
- $\zeta_{i}$ is the non-dimensional damping ratio.

The term $R_{\mathrm{hi}}$ is a non-negative real number included in the model to account for the residual contribution of higher modes to static sensitivity. In fact, from Equation (1), it follows that the static sensitivity can be expressed as:

(2) $$H\left(0\right)=\sum_{i=1}^{D}S_{i}+R_{\mathrm{hi}}$$

In this paper, Equation (1) is adopted to model the FRF of a load cell. The choice of the model complexity *D* (i.e., the number of DOFs) can be simply done by counting the modes (typically resonances) in the experimental FRF. Unlike the approach adopted in [30], where a faithful modelling in terms of masses, springs and damping is required, the approach proposed here aims to represent the sensor transfer function between input force and output readings. Basically, the force transducer characterisation entails establishing its response from 0 Hz up to the first *D* relevant modes of vibration, possibly neglecting the higher ones. In fact, the purpose of the analytical model is to represent the sensor FRF up to at least the first resonance, where the maximum frequency the force sensor can accurately measure is typically located. This is useful to evaluate the “transducer bandwidth”, which is typically estimated by setting a tolerance on the FRF amplitude with respect to static sensitivity. The bandwidth $f_{\mathrm{bw}}$ of the force sensor is calculated as the minimum frequency for which the amplitude of the FRF differs from the static sensitivity by a given tolerance tol%:

(3) $$f_{\mathrm{bw},tol\%}=\min_{f}\left(\left|\frac{\left|H(f)\right|}{\left|H(0)\right|}-1\right|\geq \frac{tol\%}{100}\right)$$

where tol% can be 1%, 5% or 10%, or any other desired value. This is consistent with Fujii’s recommendation to quantify the difference between dynamic and static response [7]. Clearly, Equation (3) points out that the bandwidth depends on the FRF shape rather than its static sensitivity. Anyway, it is worth noting that if the exact static calibration is applied to the sensor readings, then H(0)=1.

In the most general case, several modes may contribute substantially to the static sensitivity of the load cell. Alternatively, two or more relevant modes may have natural frequencies close to each other. In such cases, the FRF of the load cell needs more than a single DOF (D>1) to be accurately modelled using Equation (1). However, for the purpose of dynamic characterisation of load cells, a reasonable assumption for many applications is that the dynamic behaviour at low frequencies is dominated by the first mode, as well as the static sensitivity. In such cases, the first vibration mode is sufficient for an adequate dynamic characterisation, i.e., 1 DOF (D=1). This is the case addressed and discussed in detail in this paper, but the principles of the method presented here can also be applied to more general cases. The criterion proposed here to evaluate whether the number of DOFs *D* is adequate can be summarised as:

(4) $$\sum_{i=1}^{D}S_{i}\gg R_{\mathrm{hi}}\;.$$

In other words, the analytical model of the load cell FRF can be considered adequate when the residual contribution of $R_{\mathrm{hi}}$ to the static sensitivity is negligible.

In the next section, the experimental setup for the evaluation of the load cell FRFs with the impulsive method is described, which is the starting point of the dynamic characterisation procedure for load cells presented in this paper. Thereafter, Section 4 is dedicated to the estimation of the mode parameters of Equation (1) that optimally fit the experimental FRF by means of NLLS.

## 3. Experimental Setup and FRF Measurement

This section describes the experimental setup adopted for measuring the experimental FRF of the force transducer, which is the reference quantity for the dynamic characterisation. The technique presented in this paper is based on the impulsive excitation of the force transducer at the load application point using an impact hammer [32] equipped with an embedded piezoelectric force sensor. The minimum requirement for the reference force transducer is to have a sufficiently large and flat bandwidth. A traceable dynamic calibration of the load cell requires that the hammer has previously been calibrated with a primary method [23]. Moreover, it is worth noticing that the approach presented in this paper can be applied independently of the static calibration. In fact, the underlying assumption is that the force transducer to be characterised behaves as an LTI system in the force range considered.

**Figure 1:**
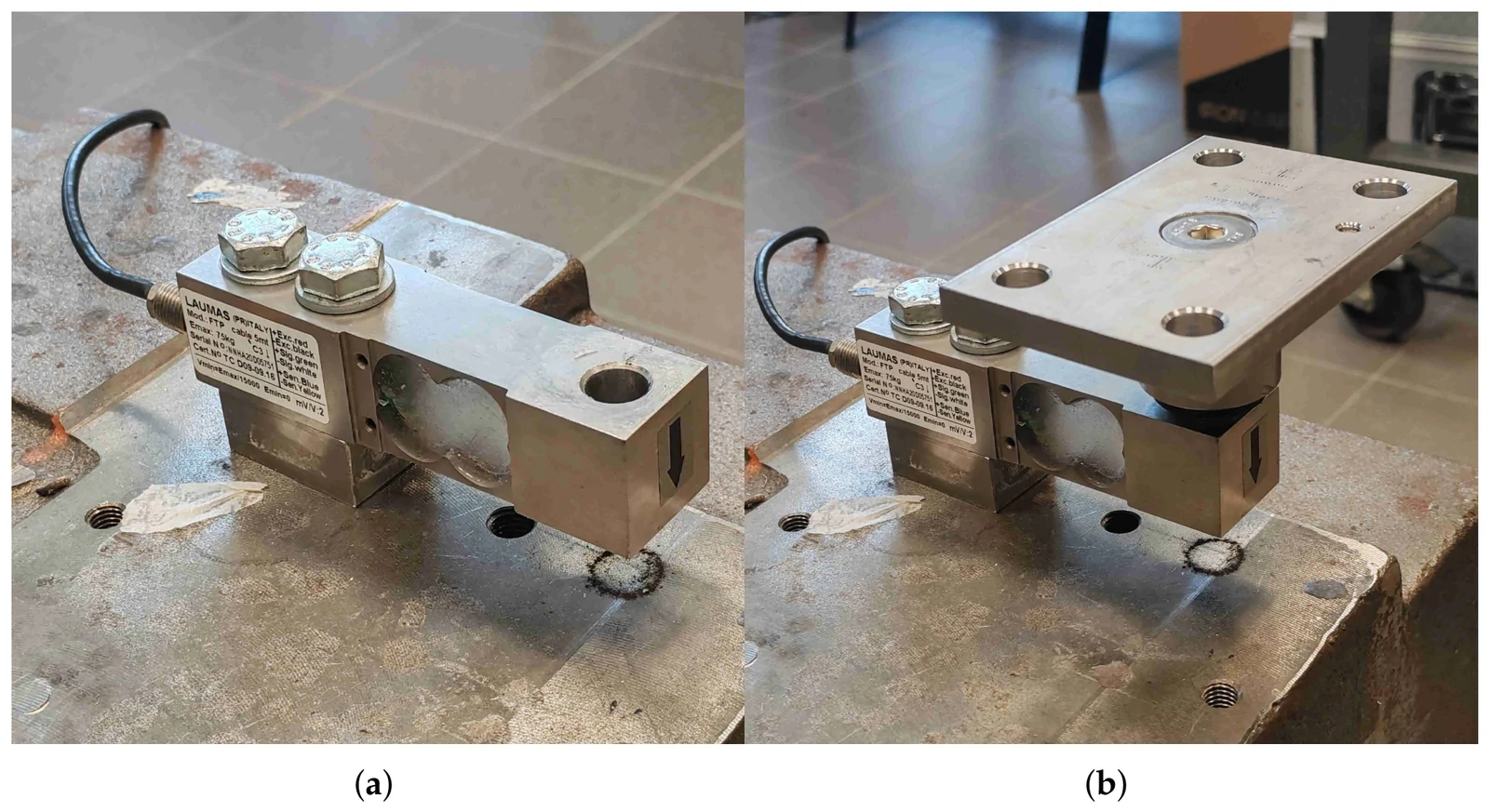
The FTP LAUMAS strain-gauge load cell fixed to a 1000 kg mass. (**a**) Load cell without accessories. (**b**) Load cell with support plate.

A single-point strain-gauge load cell is used here to demonstrate the effectiveness of the approach proposed in this paper. The load cell is an FTP LAUMAS bending-beam load cell (LAUMAS Elettronica S.r.l., Montechiarugolo, Italy; Figure 1) having a maximum measuring capacity of 75 kg, corresponding to about 735 N. The net weight of the whole sensor is 0.9 kg.

During the measurements required for the characterisation process, the transducer is fixed to a 1000 kg iron mass lying on a rubber mat on the floor. The cell is fastened to the mass using the same two screws that are used in a typical installation as suggested by the manufacturer.

### 3.1. Experimental Setup and FRF Estimation with Impulsive Method

The dynamic characterisation of a mechanical structure can be performed using the impulsive method [27,31], which has the significant advantage that all the frequencies of interest are excited at the same time, when the impact is properly realised. The free response of the load cell is recorded synchronously with the input and then processed to obtain the FRF estimate. Therefore, the FRF in the whole frequency band of interest is estimated at once.

The impact hammer used as the excitation source is a PCB Piezotronics model 086C03 (PCB Piezotronics, Inc., Depew, NY, USA) [33]. This hammer excites frequencies up to 2–4 kHz depending on the tip stiffness. A set of pre-tests varying the hammer tip was carried out in order to choose the most appropriate stiffness. The final choice for this application was the Teflon tip. Experimental aspects of impact-hammer testing are discussed in detail in [28].

**Figure 2:**
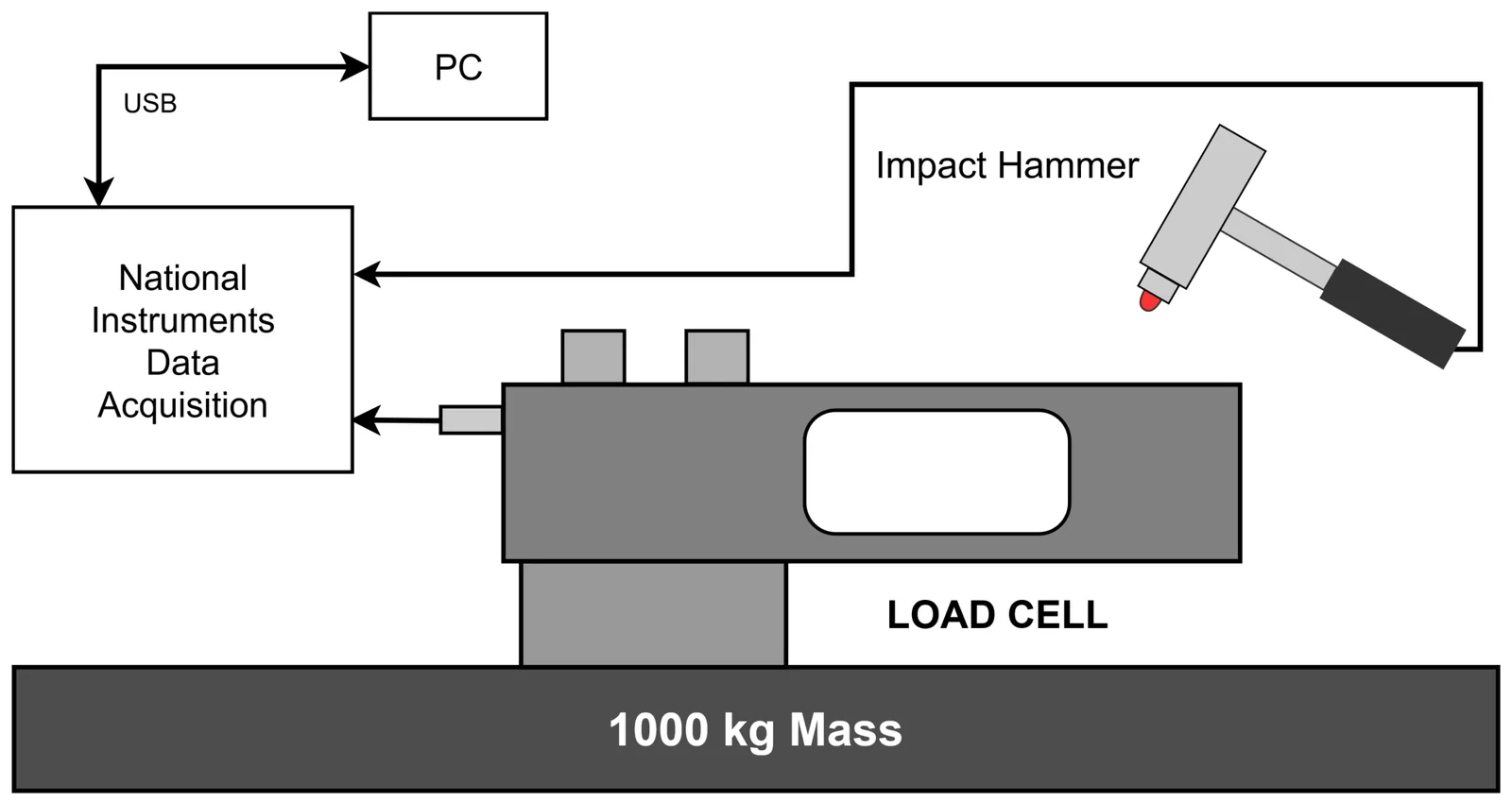
Scheme of the experimental setup for FRF measurements using the impulsive method.

All signals are acquired at a sampling frequency of 5120 Hz using National Instruments hardware (National Instruments Corporation, Austin, TX, USA) and a custom data acquisition program developed in LabVIEW (version 2024 Q1). The acquisition system consists of an NI CompactDAQ chassis connected to a PC via USB. The load cell response is measured using a National Instruments (Austin, TX, USA) NI 9218 full-bridge input module, while the hammer force signal is acquired using an NI 9234 module. All channels include built-in anti-aliasing filters. In Figure 2, a schematic diagram of the experimental setup for the dynamic characterisation of the force transducer is shown.

Since the noise is mainly on the load cell signal, i.e., the output of the LTI system, the FRF is estimated by means of the $H_{1}$ estimator defined as:

(5) $$H_{1}\left(f\right)=\frac{G_{xy}\left(f\right)}{G_{xx}\left(f\right)}$$

where $G_{xy}\left(f\right)$ is the cross-power spectrum between the load cell signal and the hammer signal, and $G_{xx}\left(f\right)$ is the auto-power spectrum of the hammer signal. The $H_{1}\left(f\right)$ estimator is a Least-Squares estimate of the system transfer function, providing an optimal estimate in the presence of noise added to the system output [31]. Since random signals are involved, such as random noise, the impact test must be repeated to estimate cross- and auto-power spectra. In the experimental tests conducted in this work, the cross-power spectrum $G_{xy}\left(f\right)$ and auto-power spectrum $G_{xx}\left(f\right)$ were estimated by averaging five repetitions of the impact test. The frequency resolution chosen for the analysis is 1 Hz. A useful measure of the quality of the FRF estimate for each frequency is the coherence $\gamma^{2}$:

(6) $$\gamma^{2}\left(f\right)=\frac{|G_{xy}{\left(f\right)|}^{2}}{G_{xx}\left(f\right)G_{yy}\left(f\right)}\;.$$

A value of coherence $\gamma^{2}\approx 1$ means that the output measured by the load cell is linearly dependent on the input signal, while values of $\gamma^{2}<1$ denote a poor signal-to-noise ratio in the output signal (e.g., at anti-resonances) or a non-linear behaviour of the system.

**Figure 3:**
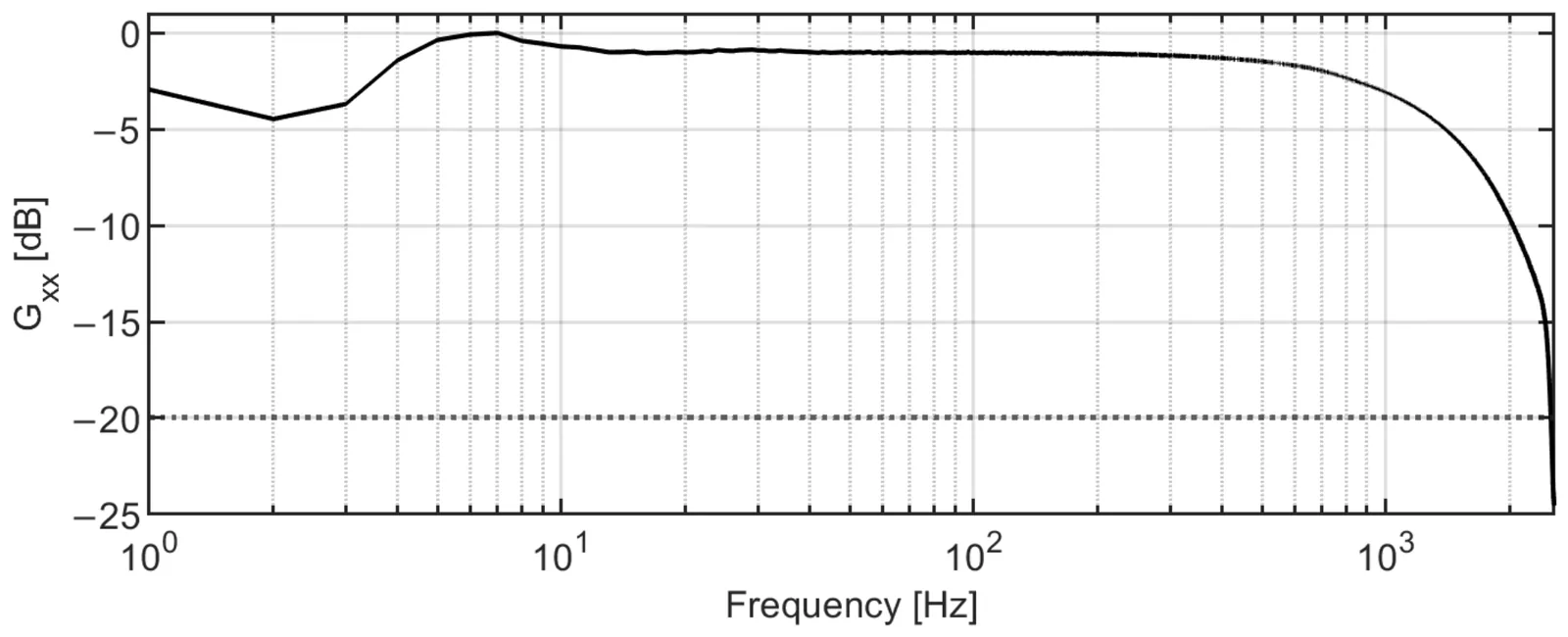
Auto-power spectrum $G_{xx}\left(f\right)$ of the input impact force. The decibel scale is referred to the maximum in the power spectrum. The dotted horizontal line indicates a 20 dB decay from the maximum, used here as a practical criterion for assessing the usable excitation bandwidth.

A final aspect to evaluate is the adequacy of the excitation force in terms of frequency content. The input power spectrum $G_{xx}\left(f\right)$ resulting from the experimental test for the load cell characterisation is shown in Figure 3. A real impact has a finite usable bandwidth, whose frequency content depends mainly on the impactor mass and tip stiffness [28,34]. As a practical rule, a decay of approximately 10–20 dB in the force spectrum over the frequency range of interest is commonly regarded as acceptable for impact testing [35]. In the present test, the input force spectrum remains within 20 dB of its maximum from approximately 5 Hz up to 2500 Hz, indicating that sufficient excitation energy is supplied over this range.

It should be stressed that this criterion concerns only the frequency content of the input excitation and does not, by itself, guarantee the quality of the estimated FRF. The latter is assessed through the coherence function $\gamma^{2}\left(f\right)$ defined in Equation (6): values close to unity indicate an adequate signal-to-noise ratio and a predominantly linear input–output relationship. Therefore, the actual frequency range employed for the FRF modelling is selected by considering both the excitation spectrum and the measured coherence.

### 3.2. Characterisation of the Experimental Mechanical Boundary Conditions

**Figure 4:**
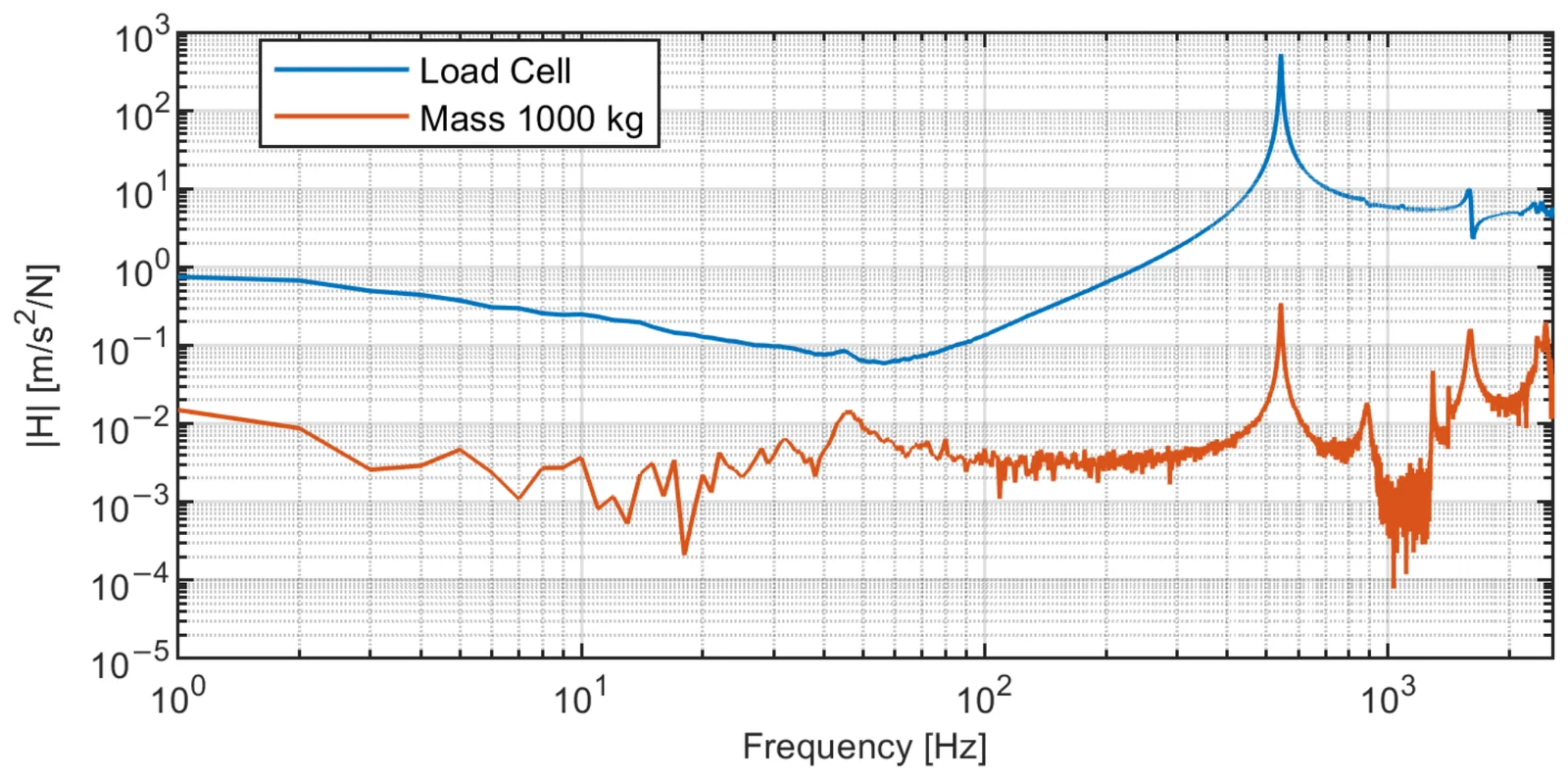
Dynamic response comparison between the 1000 kg mass and the load cell mounted on it. The FRFs are expressed in terms of acceleration over force.

When the aim is to characterise the force transducer itself, the load cell response must not be influenced by external dynamics. In such a case, it is important to evaluate the mechanical boundary conditions under which the experimental tests are conducted. Here, the load cell fastening to the 1000 kg iron mass approximates an ideal mechanical constraint. An impulsive test using the procedure described in Section 3.1 is conducted. The impact force is applied to the load point of the cell to be characterised. The response to the excitation is measured using two PCB Piezotronics 352C33 single-axis accelerometers: one is fixed on the mass, close to the cell fixing point, and the other is on the load cell close to the force application point. Figure 4 shows the FRFs resulting from the test, estimated using the procedure described in Section 3.1. The dynamics of the mass do not influence the dynamic behaviour of the force transducer, since the mass exhibits extremely low acceleration levels and resonances at higher frequencies.

## 4. Fitting Analytical Model to Experimental FRF with NLLS

The experimental measurement of the strain-gauge load cell FRF using the impulsive method represents the starting point for the dynamic characterisation procedure. However, the direct use of the experimental FRF $H_{1}\left(f\right)$ in the evaluation of the sensor bandwidth $f_{\mathrm{bw}}$ using Equation (3) involves some disadvantages. Firstly, the experimental FRF is only known at discrete points along the frequency axis, depending on the spectral resolution. Secondly, the FRF is influenced by the randomness of the estimates, especially when few averages are performed.

The model-based approach proposed in this work provides a representation of the load cell FRF using the analytical frequency domain model of Equation (1). Therefore, a crucial step of the characterisation process presented in this paper is to estimate the model parameters from experimental data by means of NLLS. The outcome is an estimate of the modal parameters of the real load cell, which is used for the analytical interpolation of the experimental FRF. This can be used to predict the load cell response to a generic input signal and to evaluate the sensor bandwidth $f_{\mathrm{bw}}$. The estimated model parameters can also be used to check the validity of the model, and for further considerations, see Section 5.

For successful characterisation, the fitting process must be carried out correctly to obtain unbiased parameter estimates. In fact, the result of the NLLS fitting depends on the shape of the measured FRF, but also on the proper setting of the fitting frequency boundaries, $f_{\mathrm{lo}}$ and $f_{\mathrm{hi}}$, and the number of DOFs *D* considered in the fitting model. Through simulated experiments, which recreate realistic scenarios, this section shows how to effectively apply NLLS fitting for dynamic characterisation purposes and provides the guidelines for adequate modelling of the experimental FRF.

### 4.1. Parameter Estimation from Experimental FRF with NLLS Fitting

NLLS fitting is used to estimate the model parameters that minimise the sum of squared residuals between the experimental FRF $H_{1}\left(f\right)$ and the generic FRF H(f) of Equation (1). The sum of squared residuals can be equivalently written in terms of Mean Squared Error (MSE):

(7) $$\mathrm{MSE}=\frac{1}{F}\sum_{f=f_{\mathrm{lo}}}^{f_{\mathrm{hi}}}{\left|H_{1}\left(f\right)-H\left(f\right)\right|}^{2}$$

where *F* is the number of points in the FRF between $f_{\mathrm{lo}}$ and $f_{\mathrm{hi}}$. Since the FRFs are complex quantities, the MSE is defined from the complex differences between experimental and fitted FRFs. Therefore, the residual minimisation involves both the real and imaginary parts of the FRF, or equivalently, amplitude and phase.

In this application of NLLS, three parameters are required from the user: lower and upper limits of the frequency range $f_{\mathrm{lo}}$ and $f_{\mathrm{hi}}$ and the number of DOFs *D* included in the model. The lower boundary of the frequency range $f_{\mathrm{lo}}$ cannot be zero due to the characteristics of the piezo-transducer embedded in the impact hammer used in the experimental measurement of the load cell FRF, which is unable to measure static loads. Therefore, the boundary must be higher than the low-frequency limitation of the reference sensor. This evaluation can also be supported by the estimated FRF coherence (Equation (6)). In fact, the coherence $\gamma^{2}\left(f\right)$ drops towards 0 Hz due to the poor signal measured in this range by the reference sensor. Therefore, the lower boundary of the fitting range should be high enough to guarantee $\gamma^{2}\left(f_{\mathrm{lo}}\right)\approx 1$. The upper limit of the frequency range for NLLS fitting $f_{\mathrm{hi}}$ and the number of DOFs *D* in the model depend on the characteristics of the load cell and cannot be defined in advance. Moreover, these two parameters have the strongest influence on the accuracy of the model and parameter estimates, and consequently, on the load cell bandwidth estimate. Therefore, in the remainder of this section, realistic simulated experiments are presented with two objectives: to determine an appropriate choice of $f_{\mathrm{hi}}$ and to show how the selected number of DOFs *D* can be validated using the condition of Equation (4).

### 4.2. Analysis of the NLLS Fitting Step with Simulated Experiments

The FRF measurement process was virtually recreated in MATLAB R2024b (The MathWorks, Inc., Natick, MA, USA) by implementing a synthetic load cell transfer function, which is based on Equation (1), with known parameters, and constitutes the ground-truth for the following analysis. The input signals employed were the same experimental signals measured in the experimental campaign for the real load cell, i.e., 5 impacts with the average auto-power spectrum depicted in Figure 3. Once the noise-free output was simulated, random Gaussian noise was added to the output to obtain an overall signal-to-noise ratio of 20 dB. The FRF was estimated from the simulated input and output signals using Equation (5) with the same parameters as the experimental tests, i.e., frequency resolution of 1 Hz and 5 averages (Section 3.1). This reproduces the randomness of an FRF estimated from a finite sample and in the presence of noise in the measured output.

**Figure 5:**
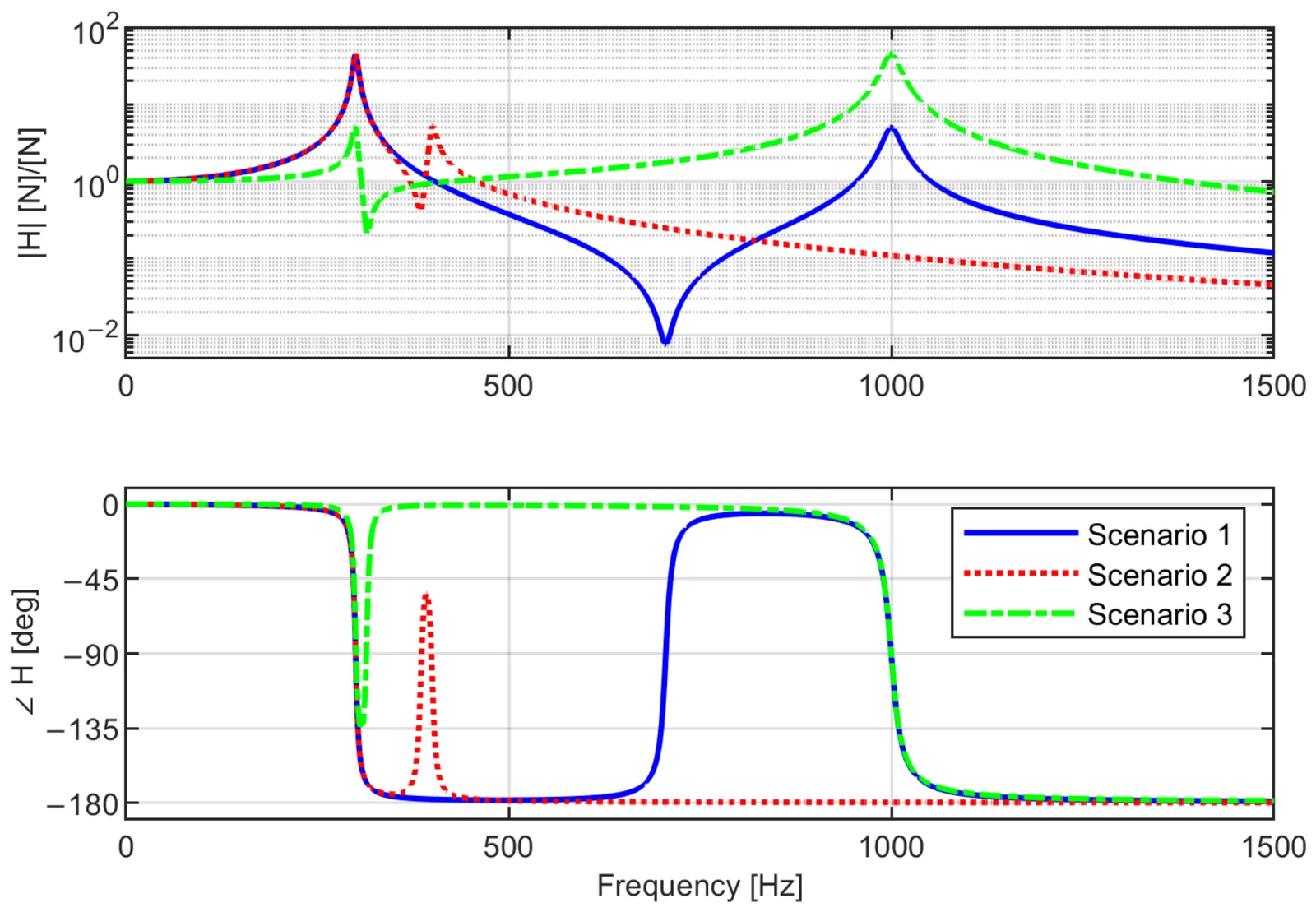
Synthetic load cell FRFs of the three scenarios considered for studying the NLLS fitting step of the measured FRF.

Three different 2-DOF FRF scenarios are considered. Their qualitative characteristics and model parameters are summarised in Table 1, while the corresponding FRFs are shown in Figure 5. The purpose of these scenarios is to study what happens in the fitted FRF when the resonances of the first two modes are sufficiently far apart not to significantly influence each other and vice versa. The scenarios also include the case in which modes neglected by the model make a significant contribution to the static sensitivity. The analysis of NLLS fitting is conducted here considering a 1-DOF model. Analogous simulations show that this analysis is also representative of cases in which a model with any number of DOFs neglects at least one mode of the actual load cell FRF.

The Monte Carlo Method (MCM) [36] was used to investigate the accuracy of the model-parameter estimates. For each trial, a new realisation of the output noise signal is generated and a new FRF estimate is computed. Once the FRF is estimated, a parametric analysis is performed by varying $f_{\mathrm{hi}}$ in the NLLS fitting, while the lower limit of the fitting range is fixed at $f_{\mathrm{lo}}=20$ Hz to reproduce a realistic condition in which the portion of the FRF close to 0 Hz cannot be estimated correctly using the piezoelectric transducer of the impact hammer. The chosen value of $f_{\mathrm{lo}}$ is conservative, since the cut-off frequencies of piezoelectric transducers are typically much closer to 0 Hz. The results of the simulated experiments are evaluated in terms of relative errors in the estimated quantities with respect to the known ground-truth values. In addition to the load cell bandwidth $f_{\mathrm{bw}}$ and static sensitivity H(0), the errors in the modal parameters $f_{n1}$, $\zeta_{1}$, and $S_{1}$, as well as in the residual sensitivity of higher modes $R_{\mathrm{hi}}$, are analysed. The reference value of $R_{\mathrm{hi}}$ is $S_{2}$ in these cases. The load cell bandwidth $f_{\mathrm{bw}}$ is computed with a tolerance of 10%. A total of $10^{3}$ trials for each scenario was considered adequate to evaluate the mean and dispersion of the errors in the quantities of interest. The dispersion is expressed as the interval between the 2.5th and 97.5th sample percentiles obtained from the MCM.

#### 4.2.1. Scenario 1: Widely Spaced Resonances with Dominant First-Mode Sensitivity

**Figure 6:**
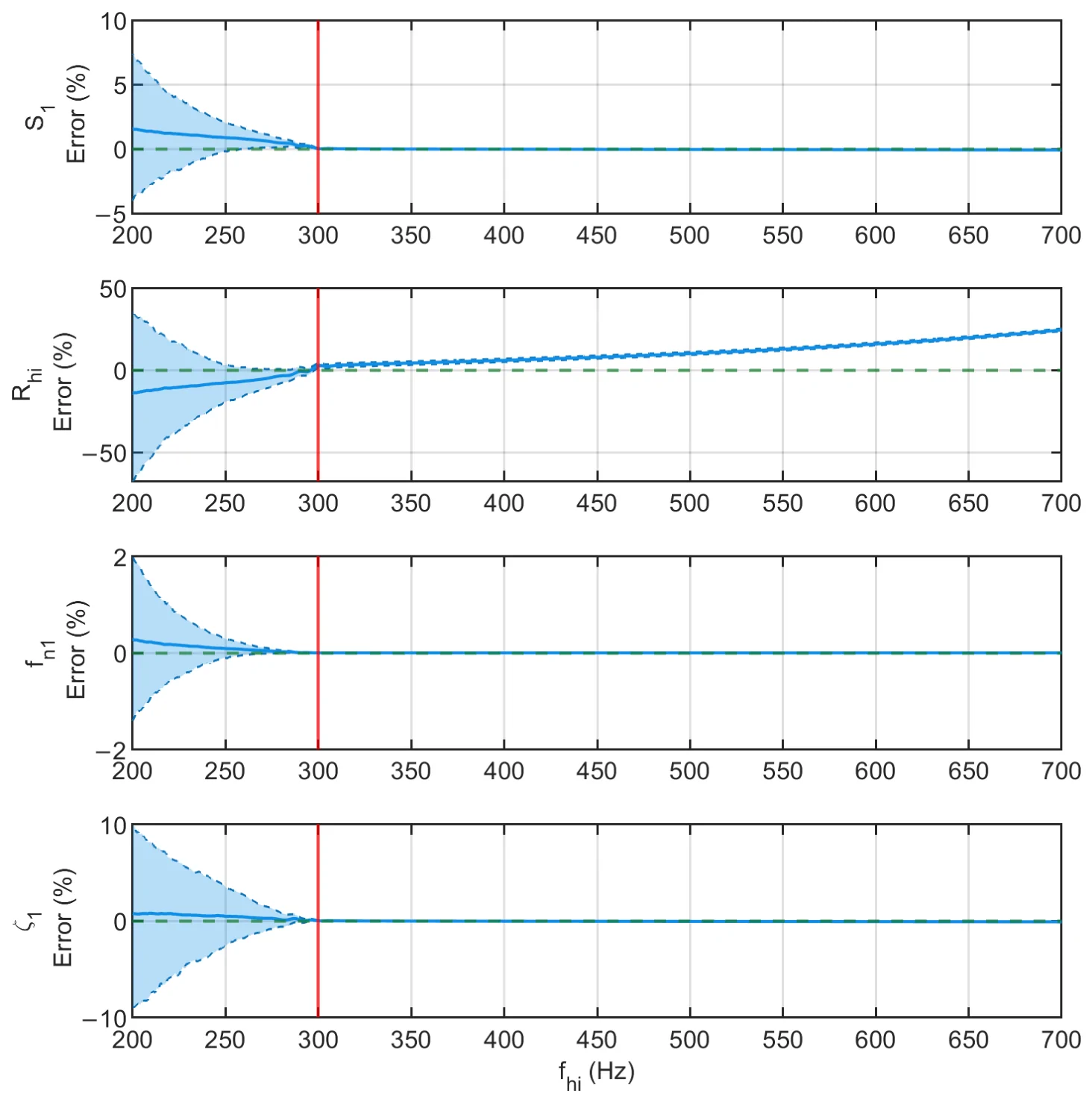
Model parameter error statistics versus $f_{\mathrm{hi}}$ obtained with MCM for Scenario 1. Solid blue curve: mean of the estimates. Dashed blue curve: dispersion of the estimates (interval between 2.5% and 97.5% sample percentiles). Red line: $f_{n1}$.

The first scenario represents a common load cell case where the higher modes do not significantly affect the first one (Figure 5). Indeed, the second resonance is far from the first one in frequency, and its static sensitivity term is significantly lower. Therefore, the effect of $R_{\mathrm{hi}}=S_{2}$ is significantly lower than $S_{1}$. Figure 6 shows the errors in the load cell parameter estimates as a function of the upper limit of the fitting frequency interval $f_{\mathrm{hi}}$, which is varied from 200 to 700 Hz (the latter approximately corresponds to the anti-resonance in the synthetic FRF). The results demonstrate an extremely high dispersion of the parameter estimations if $f_{\mathrm{hi}}$ is set below the first resonance frequency, while the estimates are more precise for $f_{\mathrm{hi}}\geq f_{n1}$. Also, the bias on the parameters related to the first mode becomes negligible when $f_{\mathrm{hi}}$ is close to or greater than $f_{n1}$. Conversely, the bias of the residual sensitivity $R_{\mathrm{hi}}$ increases as $f_{\mathrm{hi}}$ grows. Therefore, it is reasonable to state that the minimum value for the upper limit of the frequency range employed in the fitting process is approximately the first natural frequency $f_{n1}$.

**Figure 7:**
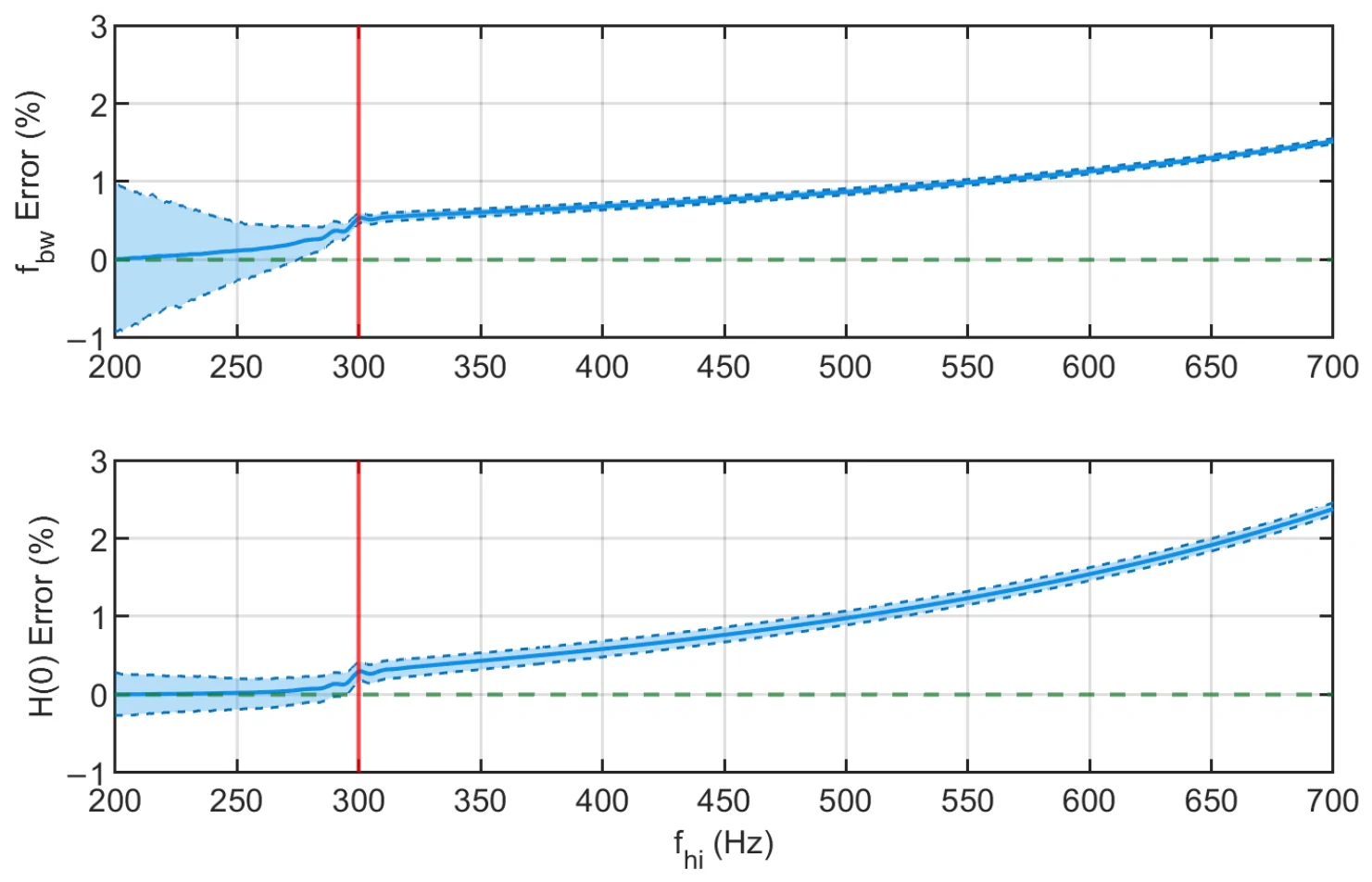
Bandwidth and static sensitivity error statistics versus $f_{\mathrm{hi}}$ obtained with MCM for Scenario 1. Solid blue curve: mean of the estimates. Dashed blue curve: dispersion of the estimates (interval between 2.5% and 97.5% sample percentiles). Red line: $f_{n1}$.

On the other hand, the errors in the estimation of $f_{\mathrm{bw}}$ and H(0) show a different trend. In Figure 7, it can be observed that the bias of the estimates decreases after $f_{n1}$ as $f_{\mathrm{hi}}$ increases.

It is important to emphasise that the estimation of dynamic parameters of the load cell is as important as that of bandwidth, since these are employed later in Section 5 for the analysis of the load cell bandwidth in operating condition. The analysis of this scenario suggests that a balanced choice is to set $f_{\mathrm{hi}}$ equal to the natural frequency $f_{n1}$, i.e., at the peak. Following this rule, the relative errors in the estimates of the first-mode parameters are lower than 0.1%, the relative error for $R_{\mathrm{hi}}$ is approximately 2.7%, while that for $f_{\mathrm{bw}}$ is lower than 0.6%.

#### 4.2.2. Scenario 2: Closely Spaced Resonances with Dominant First-Mode Sensitivity

**Figure 8:**
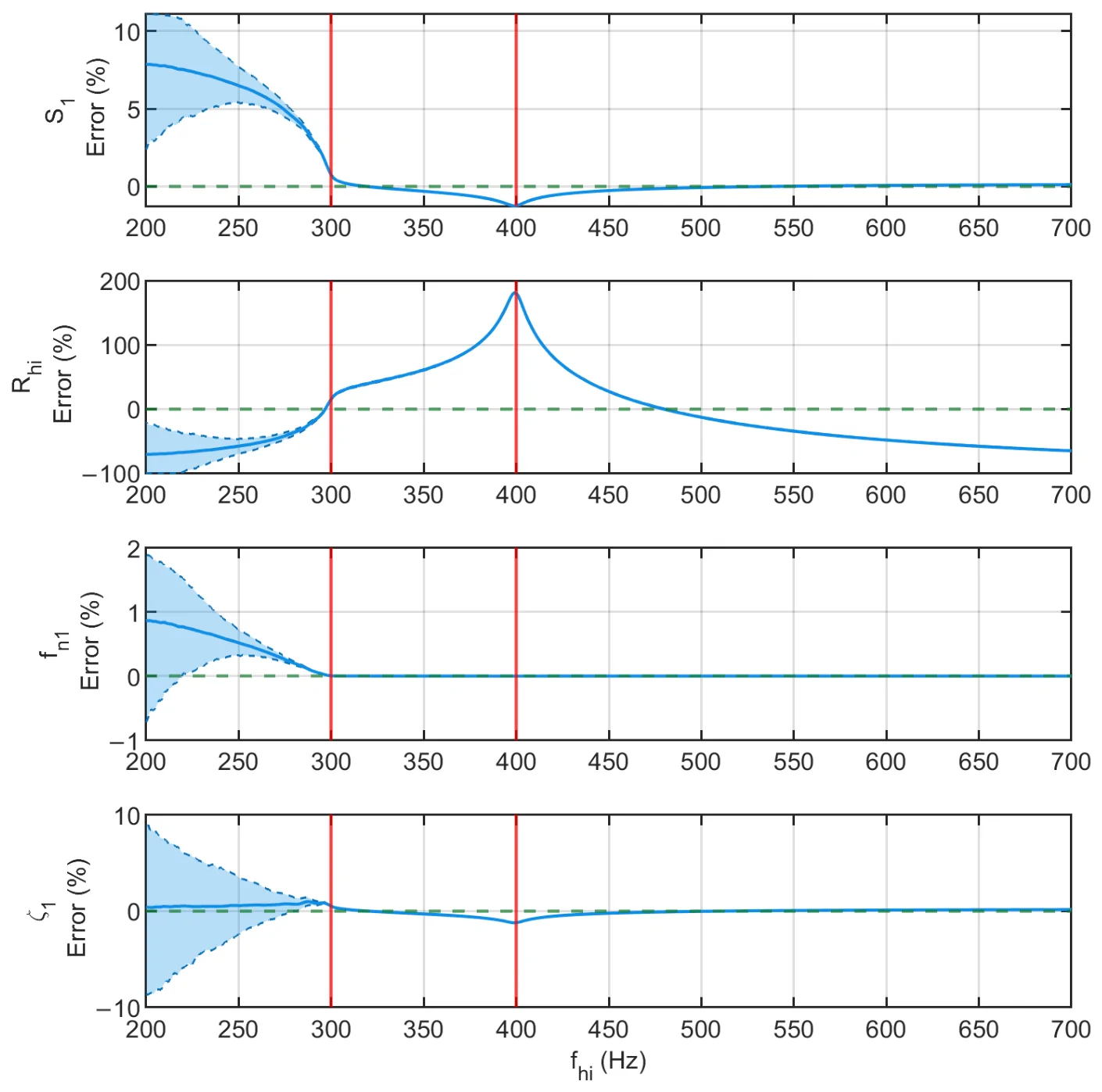
Model parameter error statistics versus $f_{\mathrm{hi}}$ obtained with MCM for Scenario 2. Solid blue curve: mean of the estimates. Dashed blue curve: dispersion of the estimates (interval between 2.5% and 97.5% sample percentiles). Red lines: $f_{n1}$ and $f_{n2}$.

**Figure 9:**
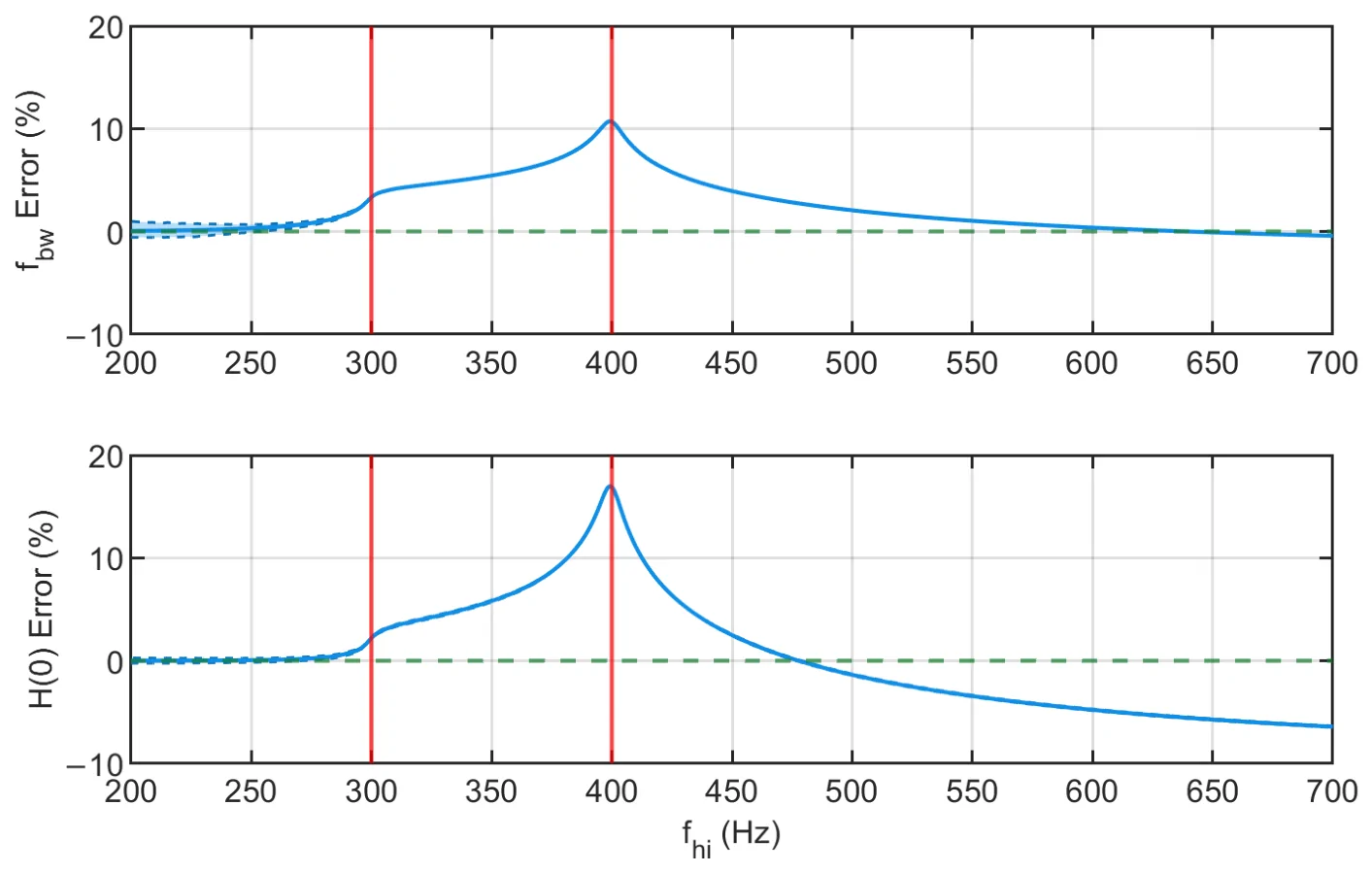
Bandwidth and static sensitivity error statistics versus $f_{\mathrm{hi}}$ obtained with MCM for Scenario 2. Solid blue curve: mean of the estimates. Dashed blue curve: dispersion of the estimates (interval between 2.5% and 97.5% sample percentiles). Red lines: $f_{n1}$ and $f_{n2}$.

The same procedure was carried out with the second scenario (Figure 5), which is the case where the higher modes (in this case, the second) influence the first more significantly in terms of the closeness of the natural frequencies, while $R_{\mathrm{hi}}=S_{2}$ can still be considered reasonably negligible. The analysis is performed using the same range for $f_{\mathrm{hi}}$ adopted for Scenario 1. Figure 8 shows the relative errors in the model-parameter estimates, while Figure 9 shows the relative errors on $f_{\mathrm{bw}}$ and H(0). The analysis of the error reveals that a severe bias in model parameters, as well as in bandwidth and sensitivity, may occur when $f_{\mathrm{hi}}>f_{n1}$. The maximum relative error occurs when $f_{\mathrm{hi}}=f_{n2}$. In fact, the 1-DOF model is no longer adequate under such conditions, since the second peak on the FRF cannot be represented by the analytical model. Therefore, even in this case, setting $f_{\mathrm{hi}}$ to the natural frequency $f_{n1}$ is a good compromise. In fact, the relative errors in the model parameters are lower than 1%, except for $R_{\mathrm{hi}}$, which exhibits a 16% error. The relative errors in $f_{\mathrm{bw}}$ and H(0) are lower than 4%. The analysis of this scenario reveals that it is still possible to retrieve an accurate estimate of all quantities needed for characterisation of dynamic behaviour when the first mode peak is weakly influenced by the higher ones in terms of the closeness of the natural frequencies.

#### 4.2.3. Scenario 3: Widely Spaced Resonances with Dominant Second-Mode Sensitivity

**Figure 10:**
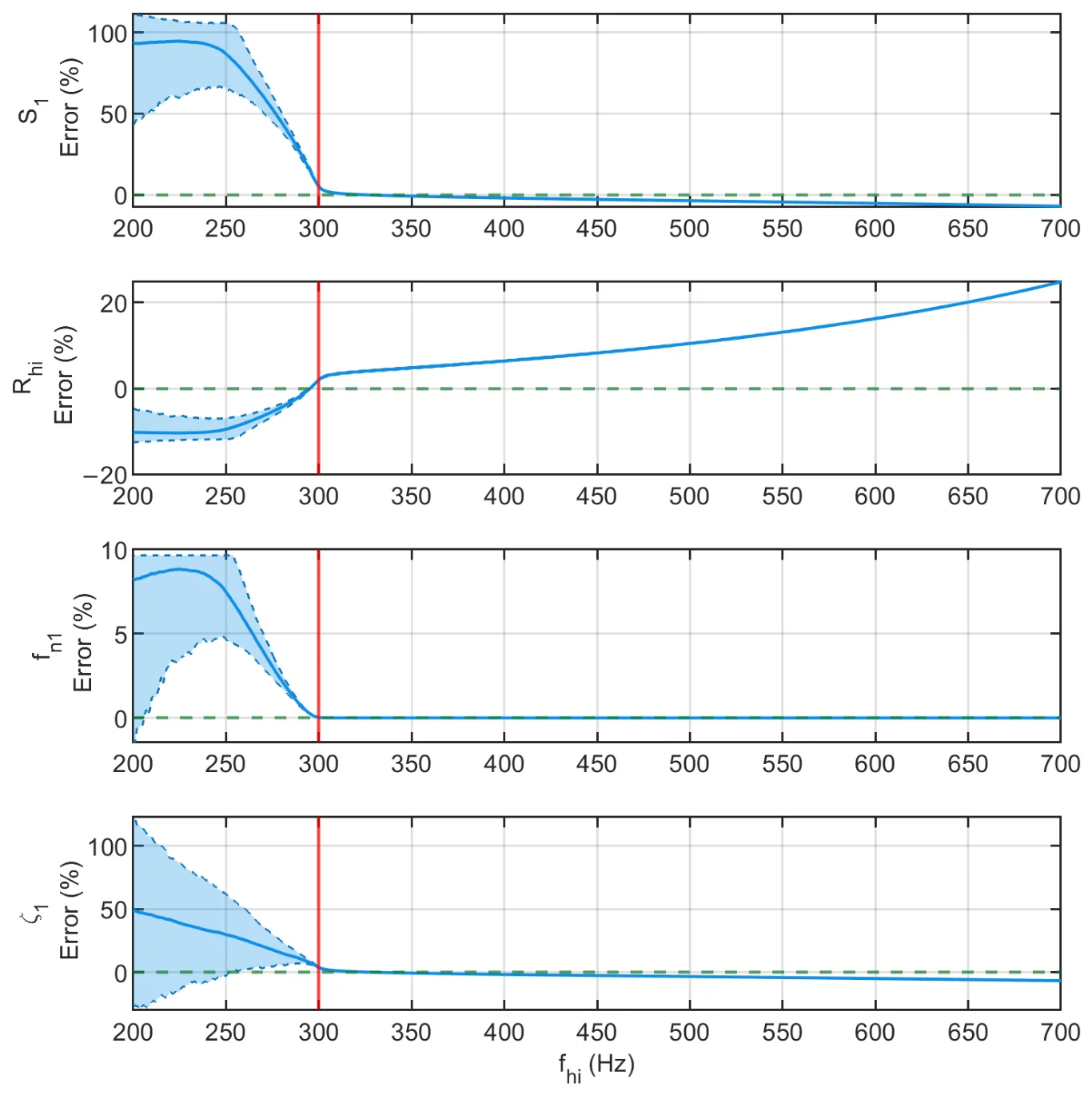
Model parameter error statistics versus $f_{\mathrm{hi}}$ obtained with MCM for Scenario 3. Solid blue curve: mean of the estimates. Dashed blue curve: dispersion of the estimates (interval between 2.5% and 97.5% sample percentiles). Red line: $f_{n1}$.

**Figure 11:**
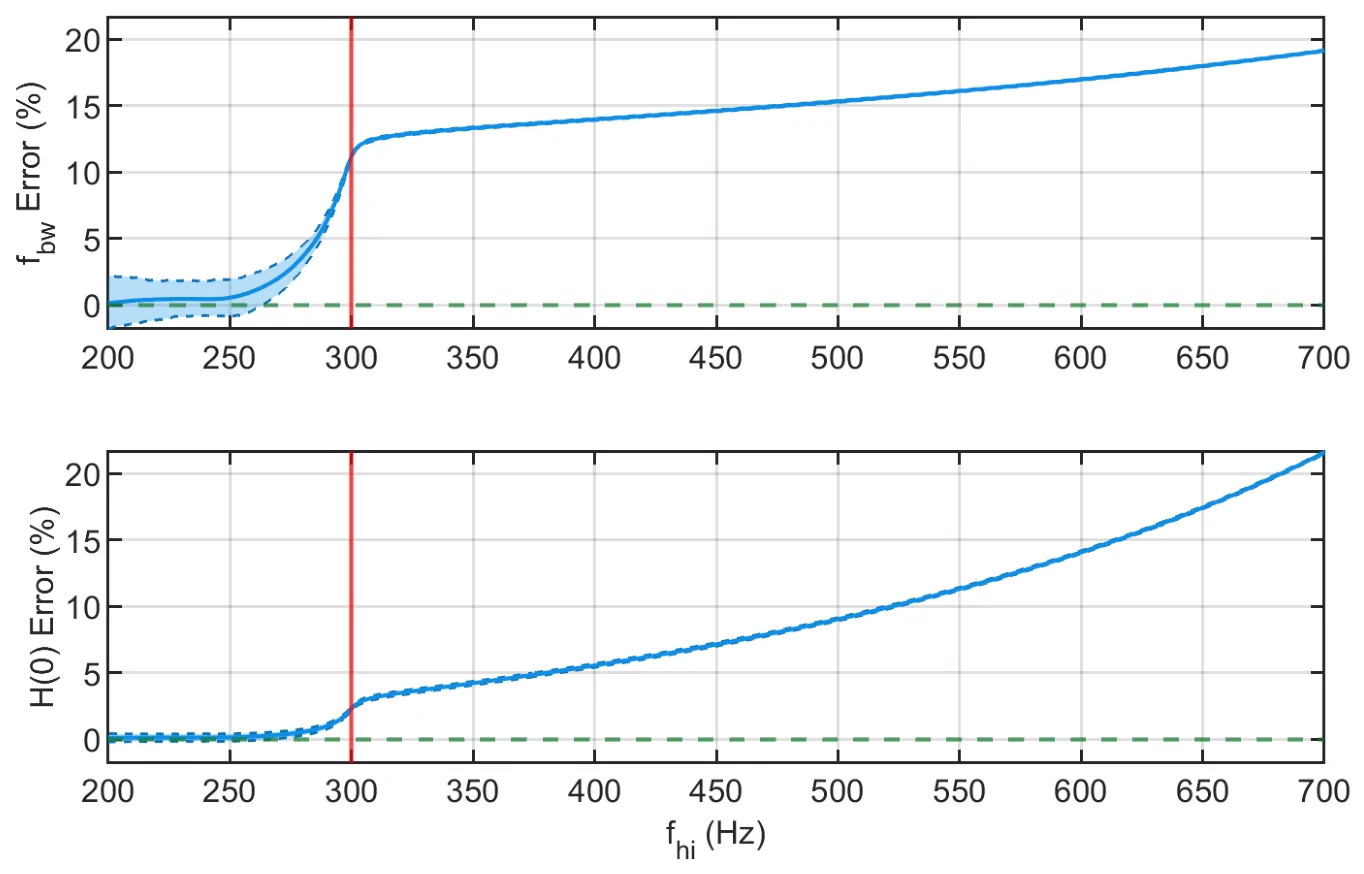
Bandwidth and static sensitivity error statistics versus $f_{\mathrm{hi}}$ obtained with MCM for Scenario 3. Solid blue curve: mean of the estimates. Dashed blue curve: dispersion of the estimates (interval between 2.5% and 97.5% sample percentiles). Red line: $f_{n1}$.

The last scenario (Figure 5) represents the case where the higher modes (in this case, the second) exhibit a greater influence on the first one in terms of static sensitivity, even though the second natural frequency is well separated from the first. In this case, the influence $R_{\mathrm{hi}}=S_{2}$ can no longer be considered negligible. The analysis is performed again using the same range for $f_{\mathrm{hi}}$ adopted for Scenario 1. Figure 10 shows the relative errors in the model-parameter estimates, while Figure 11 shows the relative errors in $f_{\mathrm{bw}}$ and H(0). Similar to the previous scenarios, when $f_{\mathrm{hi}}$ is near $f_{n1}$, the accuracy of the estimated parameters is high. When $f_{\mathrm{hi}}>f_{n1}$, the 1-DOF model becomes inadequate for the bandwidth evaluation.

Choosing $f_{\mathrm{hi}}=f_{n1}$ as in the previous analyses, the relative errors in the model parameters are below 6%. However, the error in H(0) is about 2%, while the error in $f_{\mathrm{bw}}$ is about 11%, which cannot be considered acceptable in some applications.

### 4.3. General Guidelines for NLLS Fitting in the Dynamic Characterisation of Load Cells

Two general guidelines can be drawn from the scenarios analysed, in order to guarantee an accurate modelling of the load cell FRF:

- set the upper limit of the fitting frequency range, $f_{\mathrm{hi}}$, equal to the highest natural frequency $f_{n}$ considered in the model;
- the number of DOFs *D* in the model can be considered adequate if the sensitivity residual $R_{\mathrm{hi}}$ is negligible compared to the sum of the static sensitivities $S_{i}$ of the other modes (Equation (4)).

When these two rules are followed in the modelling phase, it is reasonably guaranteed that the analytical model satisfactorily represents the experimental FRF, even if one or more DOFs are neglected. Moreover, the simulations show that a proper choice of the fitting frequency range offers the best compromise between bias and variance in the model-parameter estimates. A good approximation of the natural frequencies of the load cell is provided by the peak locations in the amplitude of the estimated FRF, since the damping ratios of these mechanical systems are typically low. Therefore, the practical application of the fitting procedure can start by setting $f_{\mathrm{hi}}$ at the first FRF amplitude peak and D=1; then, the condition of Equation (4) can be checked. If it is not satisfied, a DOF is added to the model, and $f_{\mathrm{hi}}$ is moved accordingly to the next FRF peak. If the condition is satisfied, the FRF model is adequate for the bandwidth estimation.

## 5. Procedure for Model-Based Dynamic Characterisation of Load Cells in Frequency Domain

In the previous sections, the two main building blocks of the method proposed for the dynamic characterisation of load cells were introduced and described in detail: the measurement of the experimental FRF and its modelling with the NLLS fitting. In this section, the complete procedure is presented step by step, using the load cell shown in Figure 1 as an example. The three main steps are:

1. Measurement of experimental load cell FRF using the impulsive method described in Section 3.1.
2. Modelling of experimental FRF using the NLLS fitting and the guidelines provided in Section 4.
3. Evaluation of the force transducer bandwidth from the frequency domain analytical model.

All the results are obtained and discussed using an analytical model of the load cell with a single DOF (D=1). Therefore, to improve readability, the subscript relating to the mode number is omitted from this point onwards.

### 5.1. Step 1: Measurement of Experimental Load Cell FRF Using the Impulsive Method

**Figure 12:**
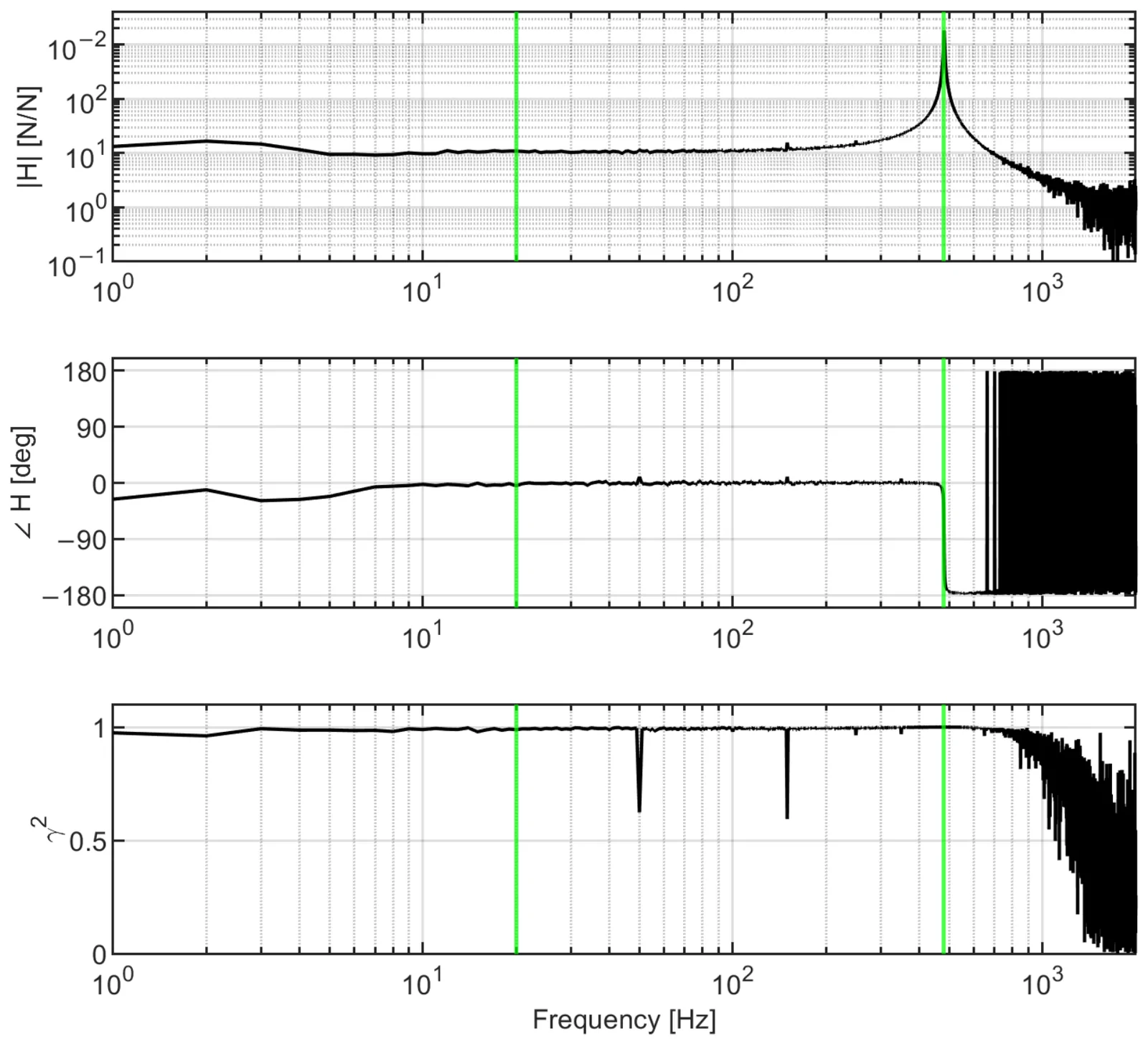
Estimated experimental FRF $H_{1}\left(f\right)$ and coherence $\gamma^{2}\left(f\right)$ of the load cell. Green lines represent $f_{\mathrm{lo}}$ and $f_{\mathrm{hi}}$ used in the NLLS fitting step.

The FRF of the load cell in Figure 1 is measured using the measurement procedure described in Section 3.1. Figure 12 shows the FRF evaluated with the $H_{1}$ estimator and the coherence $\gamma^{2}\left(f\right)$. A visual inspection of the experimental FRF reveals one clearly dominant mode at approximately 481 Hz within the frequency range where the coherence is close to unity. The resonance is identified by the pronounced peak in the FRF amplitude together with the associated phase transition from values close to $0^{\circ }$ below resonance towards $-180^{\circ }$ above it, which is consistent with the response of a lightly damped dominant mode. After the resonance, the FRF amplitude decreases rapidly. Above approximately 700 Hz, the coherence progressively departs from unity, and the experimental FRF becomes increasingly affected by measurement noise. Since the impact force spectrum remains sufficiently energetic in this frequency region (Figure 3), this loss of coherence is consistent with a reduction of the signal-to-noise ratio of the load cell output rather than with insufficient input excitation. Therefore, the FRF above this frequency is not used to draw conclusions on additional modal content. Based on the reliable portion of the measured FRF, a 1-DOF model is selected as an initial candidate, whose adequacy is subsequently verified in Step 2 through the criterion of Equation (4). The effect of the low-frequency response limitation of the piezoelectric transducer embedded in the impact hammer is not clearly visible in the FRF because of the adopted frequency resolution of 1 Hz.

### 5.2. Step 2: Modelling of Experimental FRF Using the NLLS Fitting

**Figure 13:**
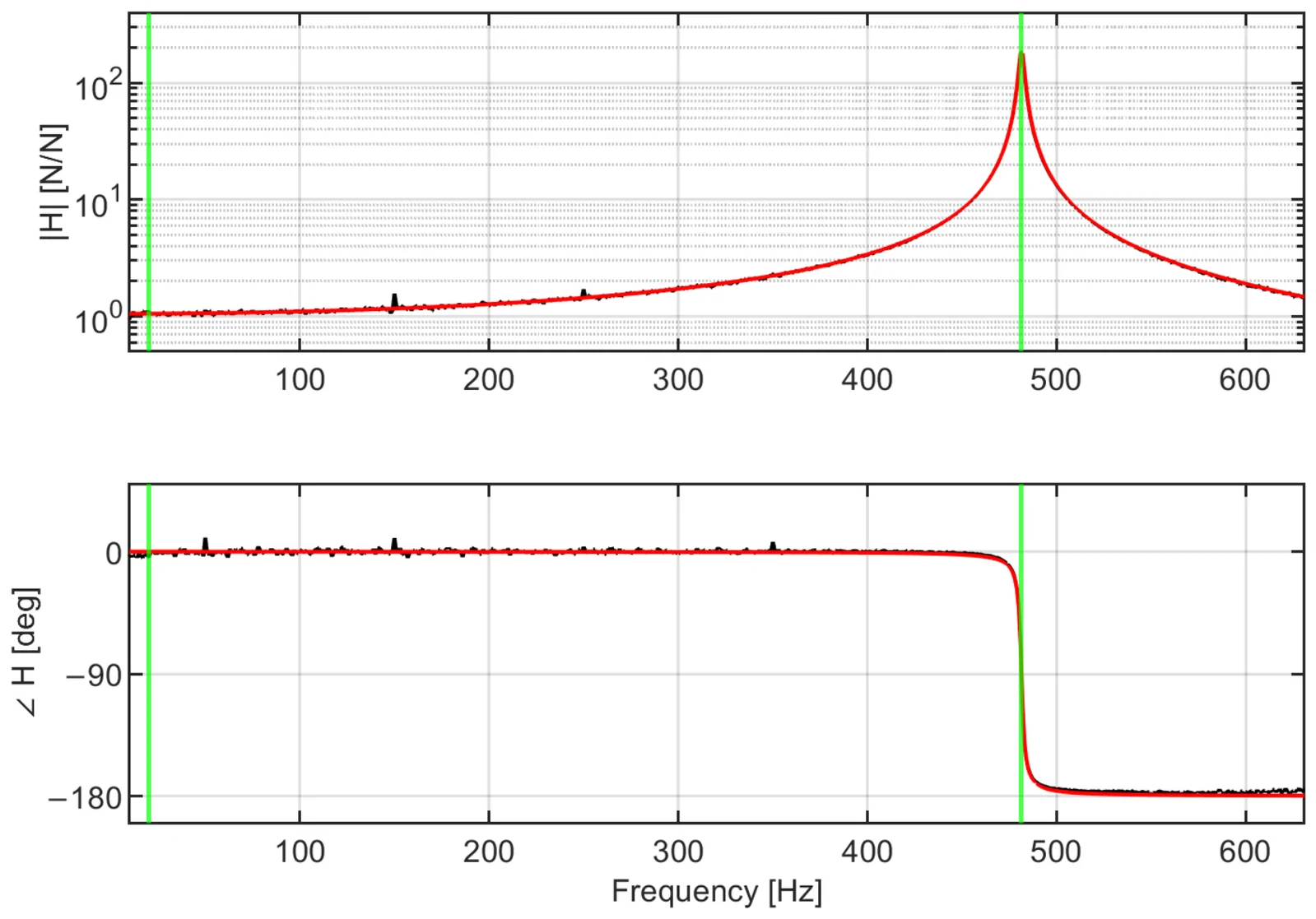
Comparison between experimental (black curve) and fitted (red curve) load cell FRFs. Green lines represent $f_{\mathrm{lo}}$ and $f_{\mathrm{hi}}$ used in the NLLS fitting step.

Once the experimental FRF of the load cell is available, the next step is frequency-domain modelling using the NLLS fitting (Section 4). Based on the single dominant resonance identified in the high-coherence portion of the measured FRF in Figure 12, a single-DOF model is selected as the initial candidate for modelling this load cell. The adequacy of the selected 1-DOF model is subsequently verified quantitatively through the residual-sensitivity criterion of Equation (4). The upper boundary for NLLS fitting is set at $f_{\mathrm{hi}}=481$ Hz following the guideline stated in Section 4. The lower boundary is set at $f_{\mathrm{lo}}=20$ Hz based on the input power spectrum of Figure 3. The coherence $\gamma^{2}\left(f\right)$ is consistently close to 1 for the whole fitting range, indicating that the noise on the output is sufficiently low and the system is linear for these frequencies. The analytical FRF resulting from NLLS fitting is depicted in Figure 13 and compared to the experimental one, while Table 2 reports the estimated parameters. The amplitude and phase computed from the analytical model faithfully represent the experimental FRF within the fitting range. The adequacy of the model is also confirmed by the condition of Equation (4), since *S* accounts for more than 99% of the static sensitivity H(0). Hence, a single-DOF model is adequate for FRF approximation and the dynamic characterisation of this sensor.

### 5.3. Step 3: Evaluation of the Force Transducer Bandwidth

**Figure 14:**
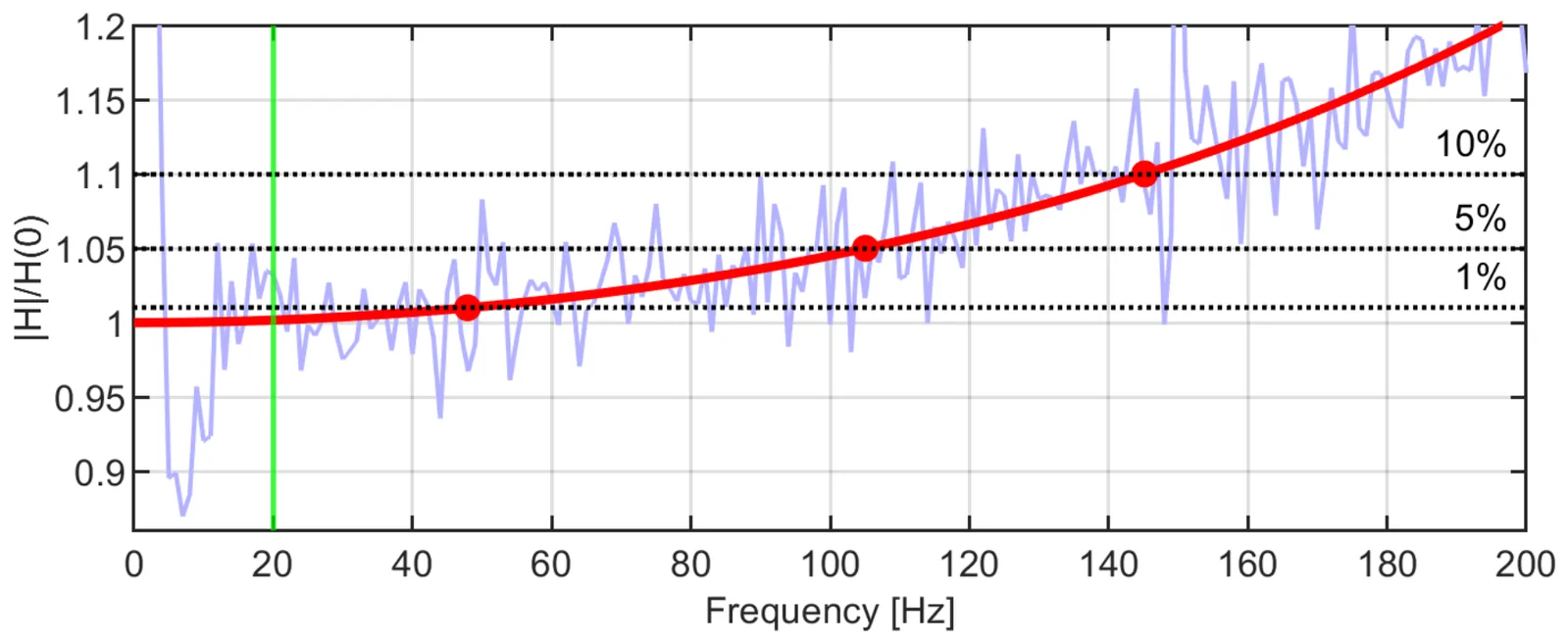
Load cell bandwidth evaluation from modelled FRF using different tolerances. Purple curve: measured FRF. Red curve: analytical FRF. Red dots: points corresponding to $f_{\mathrm{bw}}$ for different tolerances. Green line: lower bound for NLLS $f_{\mathrm{lo}}$.

At this point, the load cell bandwidth can be evaluated using the analytical model obtained in Step 2 and Equation (3) by specifying the maximum allowable deviation of the FRF amplitude from the static sensitivity. Typical tolerances suggested by the load cell supplier are: 1%, 5% and 10%. Figure 14 shows the bandwidth evaluation performed for the 1-DOF strain-gauge load cell to be characterised. The different values of $f_{\mathrm{bw},tol\%}$ are calculated by comparing the analytical FRF with the prescribed tolerances on the amplitude. The resulting values, depending on the desired tolerance, are reported in Table 3. Figure 14 depicts the analytical FRF and the experimental FRF, both normalised by the static sensitivity retrieved from the NLLS fitting. Comparing them highlights the need to derive the underlying model that generated the experimental FRF, and hence the importance of the modelling step. The use of a noisy experimental FRF in Equation (3) could lead to unreliable results. Conversely, the NLLS fitting of the model to the measured FRF reduces the influence of the random fluctuations of the FRF amplitude on the estimated bandwidth. The bandwidths evaluated under this condition refer to the load cell itself, when no mass and/or other support devices are applied, which is referred to here as the testing condition.

## 6. Validation of the Results with the Stepped-Sine Technique

**Figure 15:**
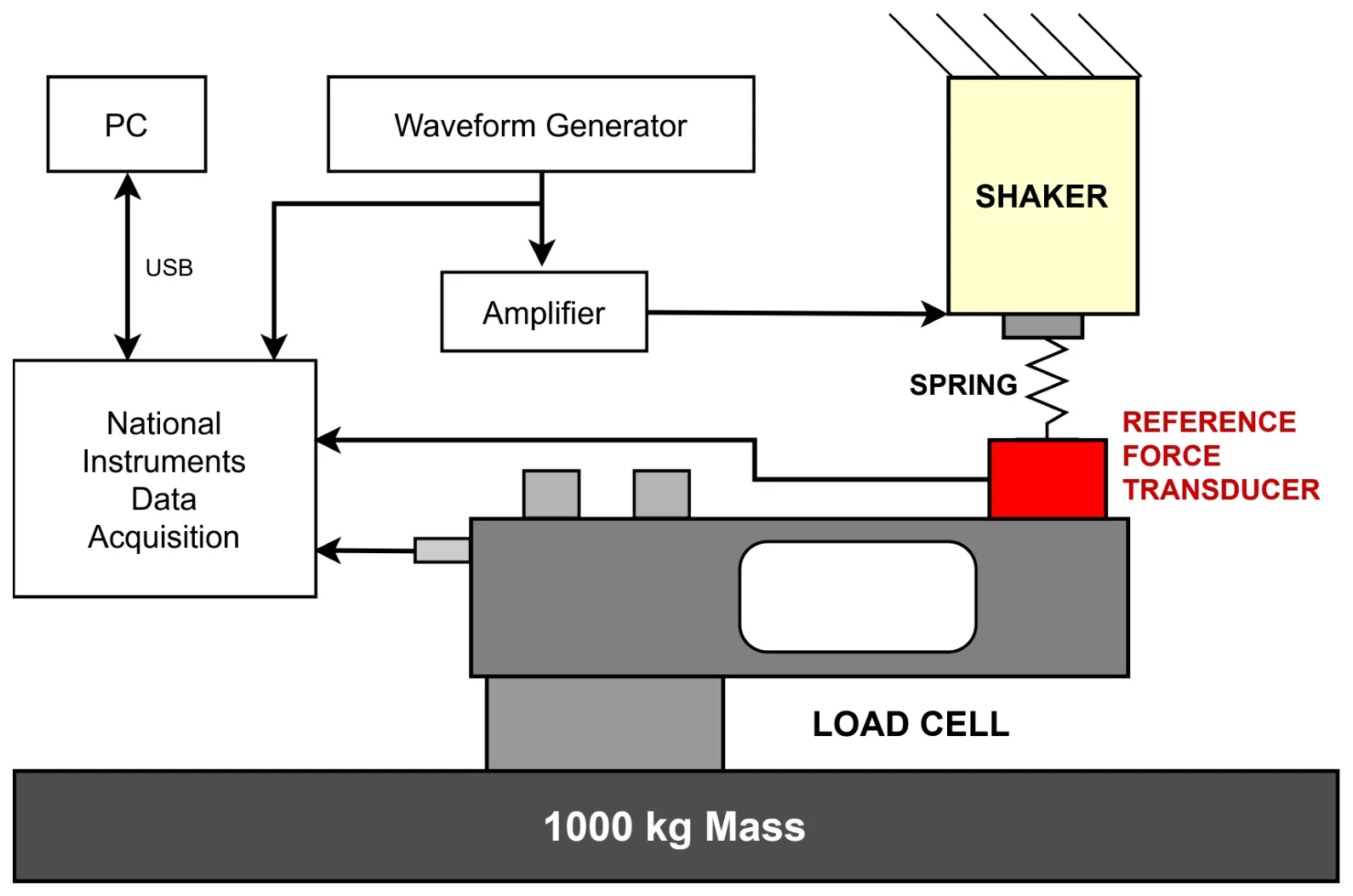
Scheme of the experimental setup for FRF measurement using sinusoidal excitation.

The modelled FRF obtained through the characterisation procedure described in Section 5 is compared here with the experimental FRF measured using an independent stepped-sine technique. The sinusoidal method, also known as the stepped-sine technique, is based on the sinusoidal excitation of the structure and returns an estimate of the FRF only at the excitation frequency $f_{\mathrm{exc}}$. Figure 15 shows a schematic of the experimental setup for the sinusoidal method.

**Figure 16:**
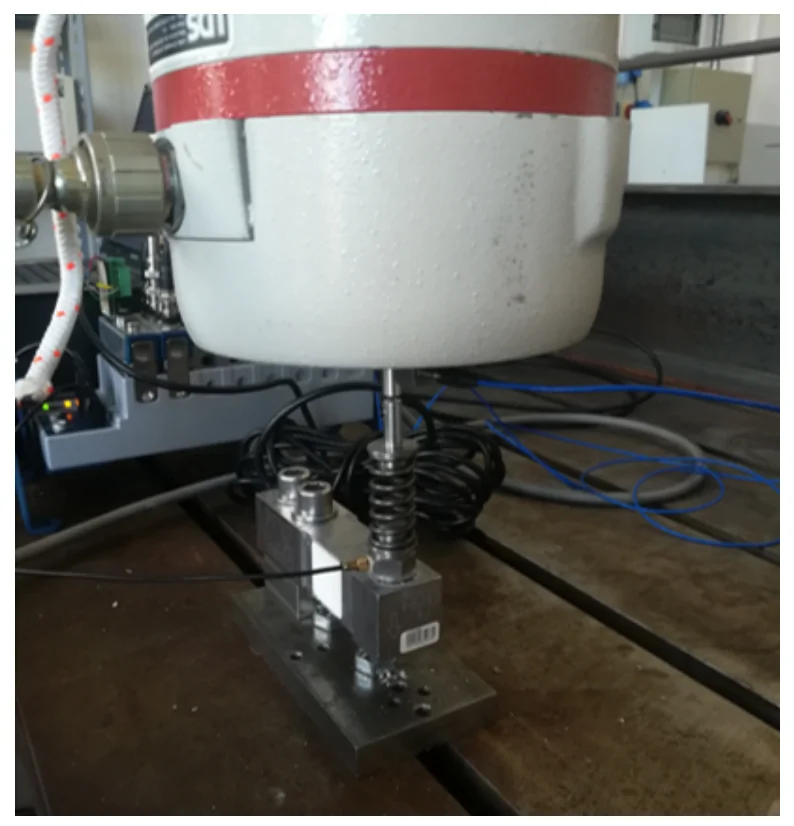
Mechanical connection between the electrodynamic shaker and the load cell under test by means of the spring and the reference force transducer.

The main advantage of the stepped-sine technique is the high signal-to-noise ratio that is achieved in the FRF estimation, since all the excitation energy is concentrated at a single frequency. Moreover, the single-frequency analysis makes it possible to reject noise components at other frequencies. However, a full-band characterisation of a load cell with the stepped-sine technique would require much longer measurement times. A reference force transducer Brüel & Kjær type 8200 (Brüel & Kjær Sound & Vibration Measurement A/S, Nærum, Denmark) [37] is bonded to the load cell measurement point and used to measure the excitation force generated by a suspended LDS V406 electro-dynamic shaker (LDS Test and Measurement Ltd., Royston, UK). A Keysight Technologies 33511B waveform generator (Keysight Technologies, Inc., Santa Rosa, CA, USA) provides the sinusoidal signal and drives the shaker through the signal amplifier. To avoid the loading effect of the actuator, a stainless-steel spring (model 13540, SODEMANN Industrifjedre A/S, Hinnerup, Denmark) connects the reference force transducer to the shaker. Figure 16 shows the mechanical connection between the shaker and load cell by means of the spring and the reference force transducer. Several levels of the spring preload were examined, and the spring preload level did not affect the dynamic behaviour of the transducer. A force with an amplitude of 10 N is chosen for the sinusoidal test due to the shaker stroke limits. Signals from the waveform generator and the reference force transducer are recorded with a voltage input board NI 9234, while the load cell bridge output is measured with an NI 9218 as in Section 3.1.

**Figure 17:**
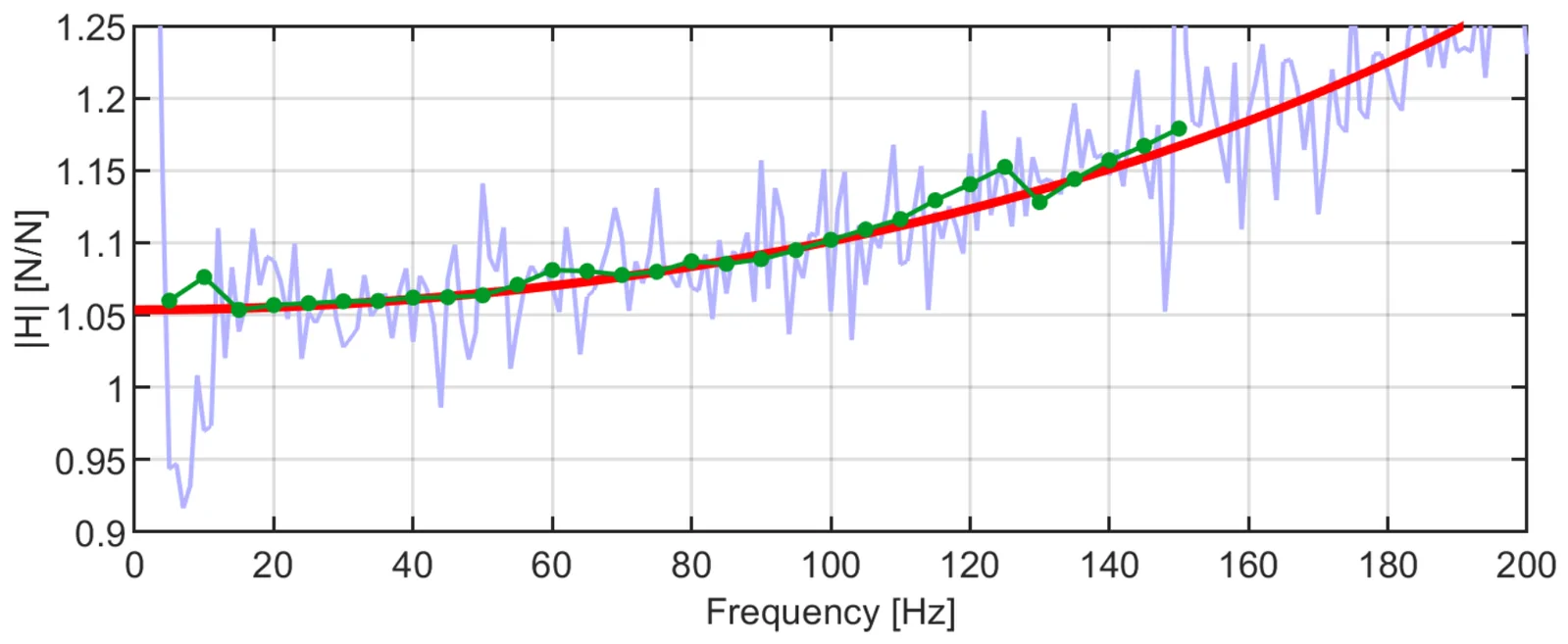
Comparison between the experimental and modelled FRFs. Purple curve: experimental FRF from impulsive test. Green dotted curve: experimental FRF from sinusoidal test. Red curve: modelled FRF using NLLS fitting and data from impulsive test.

Input and output signal spectra are computed and evaluated at the frequency $f_{\mathrm{exc}}$. To avoid leakage errors, the signals are truncated to contain an integer number of cycles. The FRF at the frequency $f_{\mathrm{exc}}$ is then estimated as follows:

(8) $$H\left(f_{\mathrm{exc}}\right)=\frac{Y(f_{\mathrm{exc}})}{X(f_{\mathrm{exc}})}$$

where *Y* is the complex spectrum of the load cell signal, and *X* is the complex spectrum of the force reference signal. The FRF estimation process was repeated for the frequency range from 5 Hz to 150 Hz with a frequency step of 5 Hz to cover the bandwidth frequency interval. Figure 17 compares the sinusoidal and impulsive experimental FRFs with the analytical FRF. The sinusoidal FRF closely follows the analytical model, confirming the reliability of the characterisation method even at low frequencies.

## 7. Evaluation of Force Transducer Bandwidth in Operating Condition

Strain-gauge load cells and force transducers are typically employed with mounting plates or junctions fixed to the load/force application point. These “support devices” enable forces to be correctly transmitted to the transducer. However, they affect the sensor dynamics, together with the boundary conditions of the installation, thus potentially resulting in a different actual bandwidth in operating conditions. Therefore, in situ dynamic calibration provides the characteristics of the sensor in its actual operating condition [26]. In some applications, in situ calibration may be possible, or even necessary, and can be performed using the same steps of the procedure described above. However, in many cases, the actual operating conditions are unknown. For example, the support device may not be known in advance, as well as the mechanical installation of the sensor. Therefore, the load cell alone must be characterised in the laboratory on a test bench such as the one described in Section 3.1.

**Figure 18:**
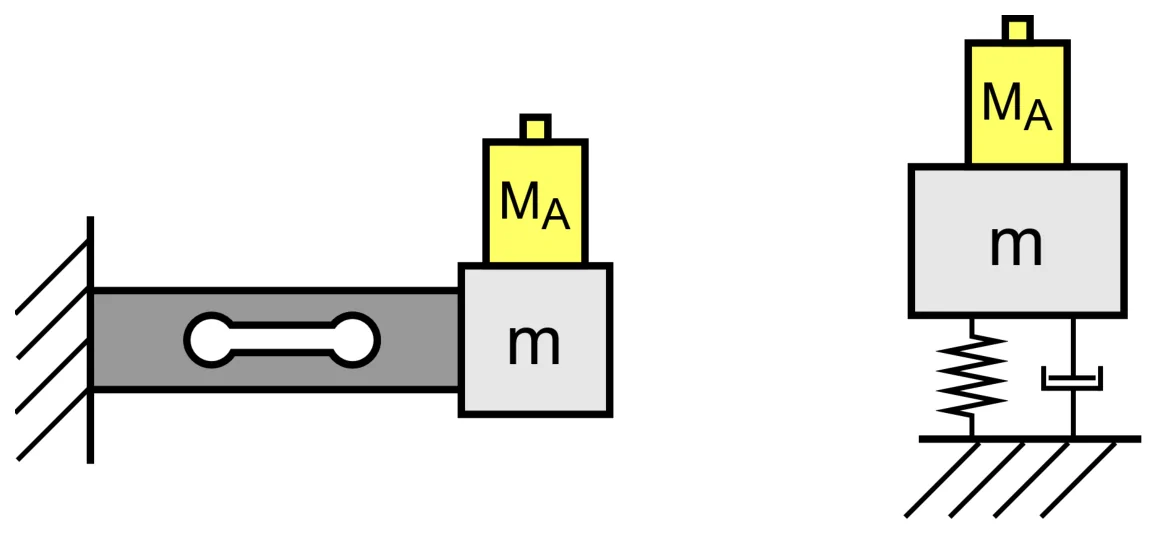
Load cell and attached mass $M_{A}$ forming a 1-DOF combined system, which can be represented as a mass-spring-damper system.

An example is the load cell characterised in Section 5. In such a case, it is useful to estimate the effect of an applied mass on the sensor dynamics and, in particular, on its actual bandwidth. An approach analogous to [16] can be adopted when the load cell can be modelled as a 1-DOF system, and the operating conditions can be represented by an additional mass $M_{A}$ rigidly attached to the sensor (e.g., a load mass or the mass of a support device). The schematic of the combined system is shown in Figure 18.

In this context, the total mass of a single MSD system can be split into two contributions:

- *m* is the mass of the load cell participating in the vibration mode (often referred to as “head mass” [14]);
- $M_{A}$ is the mass applied at the measuring point of the load cell.

When a mass $M_{A}$ is applied at the end of the load cell, the participating mass changes from *m* to $m+M_{A}$, and the natural frequency $f_{n}$ (or equivalently the angular frequency $\omega_{n}$) of the whole system changes accordingly as [6,20]:

(9) $$\omega_{n}=2\pi f_{n}=\sqrt{\frac{k}{m+M_{A}}}\;,$$

where *k* is the equivalent stiffness of the load cell, and $m+M_{A}$ represents the total equivalent mass of the mass-spring-damper system [27,31]. Using Equation (9) and the values of *k* and *m*, the natural frequency $f_{n}$ of the load cell can be predicted as a function of different applied masses $M_{A}$.

To estimate the two unknowns *k* and *m*, further impact tests can be performed using different masses $M_{A}$ attached to the load cell. From the measured FRFs, the natural frequency $f_{n}$ can be estimated using the NLLS fitting method described in this paper. Each test conducted is denoted by the subscript *t* for a total of *T* different masses attached to the load cell being characterised, thus returning a set of pairs $\left(M_{A,t},\omega_{n,t}\right)$. Using Equation (9), an equation for each test conducted can be written, resulting in the following linear system:

(10) $$\left[\begin{array}{ccc} 1 & \; & -\omega_{n,1}^{2}\\ \; & \vdots & \\ 1 & \; & -\omega_{n,t}^{2}\\ \; & \vdots & \\ 1 & \; & -\omega_{n,T}^{2} \end{array}\right]\left[\begin{array}{c} k\\ m \end{array}\right]=\left[\begin{array}{c} M_{A,1}\;\omega_{n,1}^{2}\\ \vdots \\ M_{A,t}\;\omega_{n,t}^{2}\\ \vdots \\ M_{A,T}\;\omega_{n,T}^{2} \end{array}\right]$$

When T≥2, the parameters *k* and *m* can be estimated from this set of equations using, for example, the Ordinary Least-Squares (OLS) method. Thereafter, the natural frequency in operating conditions can be computed as a function of the applied mass using Equation (9) and substituted into Equation (1) to predict the actual bandwidth $f_{\mathrm{bw}}$ of the sensor.

**Figure 19:**
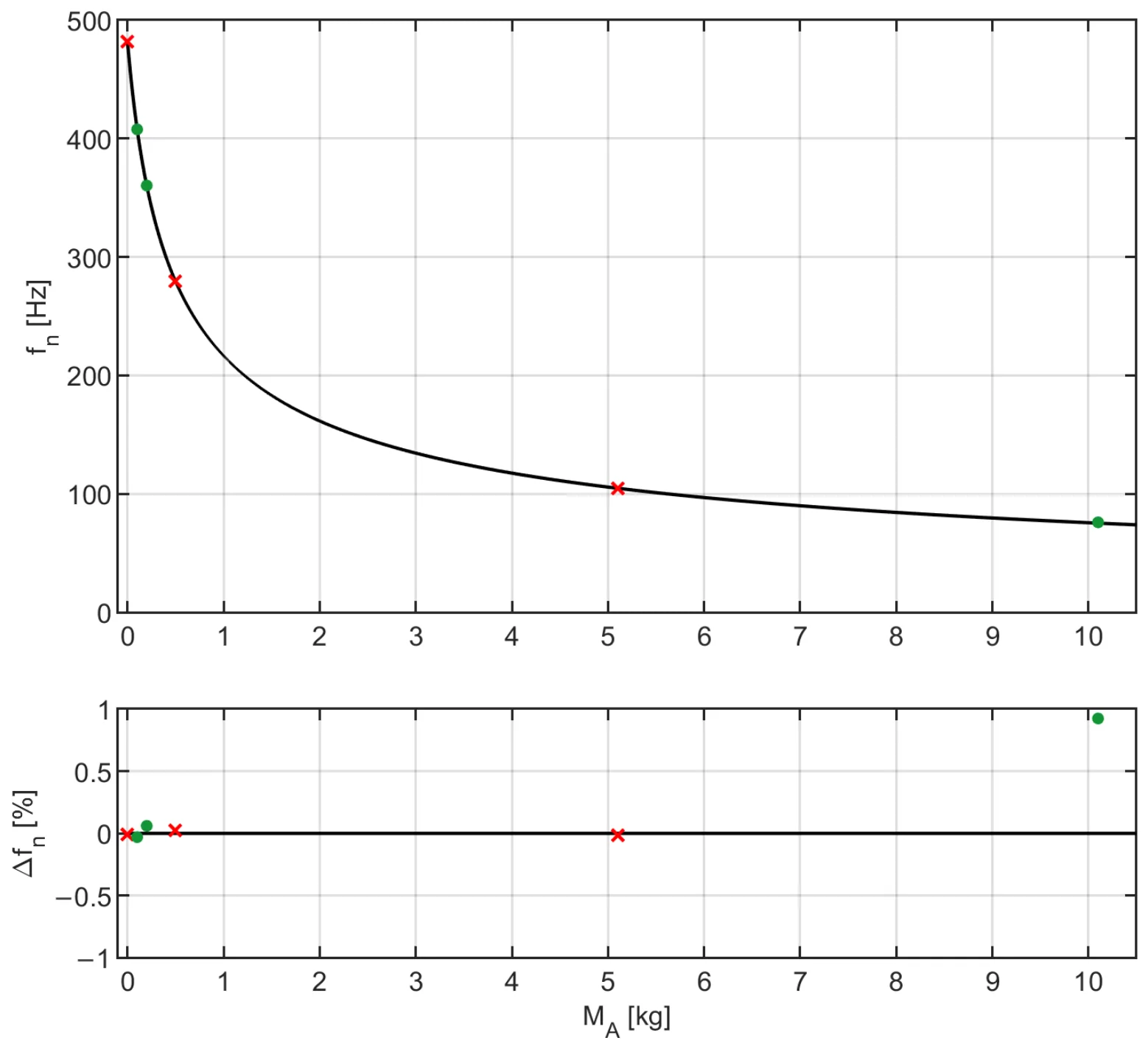
Natural frequency $f_{n}$ of the load cell as a function of the applied mass $M_{A}$: comparison between experimental results and the model with estimated parameters. Red crosses: data used to estimate *k* and *m*. Green dots: data used for prediction accuracy evaluation.

This method has been applied to the load cell used as the example in the previous steps (Figure 1). The FRF estimated in the testing condition consists of the special case of Equation (9) when $M_{A}=0$, i.e., without applied masses. Therefore, an equation of the linear system of Equation (10) is already available. Further FRFs are measured using the added masses 0.1 kg, 0.2 kg, 0.5 kg, 5.0 and 10.0 kg. For the latter two masses, the mass of the support (0.1 kg) must be added. The natural frequency for each measured FRF is estimated using the NLLS approach described in this paper. All the resulting data are summarised in Table 4. The pairs of applied mass and natural frequency $\left(M_{A,t},\omega_{n,t}\right)$ are split into two groups. The masses 0.0 kg, 0.5 kg and 5.1 kg are used in Equation (10) to estimate the unknown parameters *k* and *m*, while the others are used to verify the accuracy of the predictions. Figure 19 shows the result of the fitting and the relative error in the prediction of the load cell natural frequency $f_{n}$. The values resulting from the OLS are k= 2322 N/mm and m= 0.254 kg (about 28% of the whole transducer mass).

**Figure 20:**
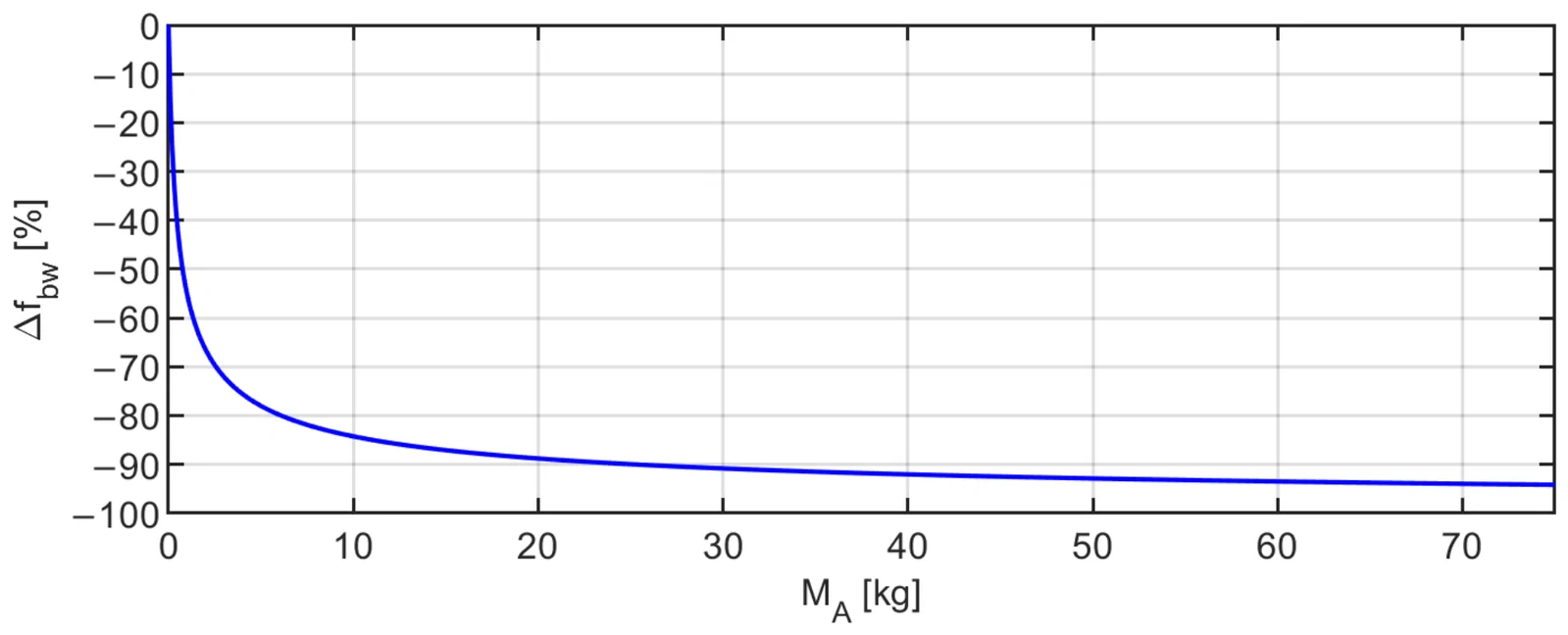
Predicted load cell bandwidth $f_{\mathrm{bw}}$ reduction depending on the applied mass $M_{A}$. This is valid for any value of the tolerance tol% considered (1%, 5% and 10%).

The results show that this method is very effective in predicting variations of the natural frequency of the load cell caused by an applied mass. In fact, the relative error is below 0.1% for the data used for fitting and less than 1% in the worst case for the data left for validation. The example reported here shows that using only a few added masses $M_{A}$ to estimate *k* and *m* is sufficient to retrieve a reasonably accurate law to predict the actual $f_{n}$ for a given $M_{A}$. Clearly, the number of tests *T* can be increased to improve the reliability of parameter estimates and predictions. Once the actual natural frequency $f_{n}$ is calculated, the value can be substituted into the synthetic FRF to predict the actual transducer bandwidth. The effect of damping on the bandwidth can be neglected for low damping ratios (e.g., ζ<10%), since it only weakly affects $f_{\mathrm{bw}}$. The predicted variation of $f_{\mathrm{bw}}$ can be plotted against $M_{A}$ so as to obtain a useful chart for the use of the sensor in operating conditions (Figure 20).

## 8. Conclusions

This paper presented a model-based methodology for the dynamic characterisation of load cells and force transducers, designed to maintain experimental and methodological simplicity while accounting for metrological considerations. The proposed approach relies on impact testing using an instrumented modal hammer to estimate the FRF under the assumption that the transducer behaves as an LTI system. Working in the frequency domain offers clear advantages when estimating the experimental transfer function. The FRF can be estimated through averaging, while the coherence function provides an indicator of the quality of the estimate. The experimental FRF is subsequently represented by a single- or multi-DOF frequency-domain analytical model, whose parameters are estimated via NLLS. By contrast, in [6], the fitting step for model-parameter estimation is performed in the time domain, resulting in a more complex procedure that is more sensitive to noise.

Another key operational advantage of the method is its minimal demand on the user. In fact, unlike [6,30], the modelling does not explicitly require the definition of a physical model in terms of masses, stiffnesses, and damping parameters; the user is only required to select the number of DOFs *D* to include in the transfer function. This choice can be performed straightforwardly either through visual inspection of the experimental FRF by counting the resonant peaks or systematically by increasing *D* until the higher-mode residual static sensitivity, $R_{\mathrm{hi}}$, becomes negligible compared to the total static sensitivity H(0) (i.e., $\sum_{i=1}^{D}S_{i}\gg R_{\mathrm{hi}}$).

To ensure unbiased and robust parameter identification, an empirical rule for selecting the NLLS fitting frequency range was formulated and validated. Extensive numerical investigations using the MCM across different scenarios demonstrated that setting the upper bound of the fitting interval, $f_{\mathrm{hi}}$, at the natural peak frequency ($f_{ni}$) of the highest mode included in the model offers the optimal compromise between bias and variance in the estimation of model parameters, static sensitivity, and transducer bandwidth.

Once the analytical FRF is identified, the dynamic bandwidth of the sensor $f_{\mathrm{bw}}$ can be evaluated for any specified amplitude deviation tolerance, bypassing the noise-induced fluctuations inherent to discrete experimental spectra. Furthermore, for transducers characterised by a single dominant mode, the analytical framework enables the extrapolation of the actual operational bandwidth as a function of an external mass $M_{A}$ attached to the sensor.

Furthermore, deriving an analytical FRF model makes it possible to simulate the dynamic output response of the force sensor to an arbitrary time-varying signal. This can be useful for estimating deviations from static characteristics as suggested in [7].

The proposed methodology was experimentally demonstrated on a strain-gauge bending-beam load cell, yielding an identified first natural frequency of 481.4 Hz. Based on the fitted analytical model, the dynamic bandwidths were evaluated as 47.9 Hz, 105.1 Hz, and 154.2 Hz for allowable deviations from static sensitivity of 1%, 5%, and 10%, respectively. Independent stepped-sine measurements confirmed the accuracy of the fitted analytical model, particularly in the low-frequency region where piezoelectric impact sensors exhibit physical limitations. For single-DOF configurations, a simplified mass-spring framework successfully predicted the effect of an added mass $M_{A}$ under operating conditions, with a worst-case relative error below 1% in the prediction of the natural frequency.

While the method was experimentally demonstrated and validated on a single type of strain-gauge bending-beam load cell, the underlying mathematical formulation is general and intrinsically applicable to a wide variety of force transducers. However, certain limitations must be taken into account:

1. LTI assumption: The transducer must not exhibit significant non-linearities within the operating range; otherwise, the LTI system hypothesis becomes non-representative.
2. Impact accessibility and alignment: The force application point must be physically accessible with the reference hammer. Angular misalignments between the impact direction and the sensor measurement axis during calibration introduce transfer function estimation errors and operational uncertainty.
3. Spatial dimensionality: The characterisation is limited to a single measurement axis. Because force transducers are three-dimensional elastic bodies, unmodelled off-axis or complex spatial vibration modes may induce model discrepancies.

Future research should focus on:

1. Evaluating the performance of the proposed method across different types, capacities and geometries of force transducers in order to validate the generality of the proposed approach.
2. Quantifying the contribution of impact misalignments to FRF uncertainty and assessing the need to mitigate human variability through the implementation of automated impulse force generation systems.
3. Developing a comprehensive metrological uncertainty evaluation and budget for the complete dynamic characterisation process of the force transducer, including the evaluation of parameter estimation uncertainties in the analytical FRF model.

## Figures and Tables

**Table 1:** Qualitative characteristics and dynamic model parameters of the three 2-DOF synthetic load cell scenarios considered in the simulated experiments.

Scenario	Characteristics	$S_{1}$	$f_{n1}$ [Hz]	$\zeta_{1}$	$S_{2}$	$f_{n2}$ [Hz]	$\zeta_{2}$
1	Widely spaced resonances, $S_{1}>S_{2}$	0.9	300	0.01	0.1	1000	0.01
2	Closely spaced resonances, $S_{1}>S_{2}$	0.9	300	0.01	0.1	400	0.01
3	Widely spaced resonances, $S_{1}<S_{2}$	0.1	300	0.01	0.9	1000	0.01

**Table 2:** Dynamic model parameters of the load cell under characterisation estimated with NLLS.

*S*	ζ	$f_{n}$	$R_{\mathrm{hi}}$
1.054	0.27%	481.4 Hz	$7.4\cdot 10^{-8}$

**Table 3:** Strain-gauge load cell bandwidth resulting from the dynamic characterisation. The tolerance refers to the sensor FRF amplitude.

Tolerance	1%	5%	10%
$f_{\mathrm{bw}}$ [Hz]	47.9	105.1	154.2

**Table 4:** Natural frequencies $f_{n}$ of the 1-DOF load cell estimated from experimental FRFs with NLLS versus the added masses $M_{A}$.

$M_{A}$ [kg]	$f_{n}$ [Hz]
0.0	481.4
0.1	407.6
0.2	360.2
0.5	279.4
5.1	104.8
10.1	76.1

## Data Availability

The raw data supporting the conclusions of this article will be made available by the authors on request.

## Abbreviations

The following abbreviations are used in this manuscript:

DOF	Degree of Freedom.
FRF	Frequency Response Function.
LDI	Laser-Doppler Interferometry.
LTI	Linear Time-Invariant.
MCM	Monte Carlo Method.
MSD	Mass-Spring-Damper.
NLLS	Non-Linear Least-Squares.
OLS	Ordinary Least-Squares.
